# Supplementary material for: Global Landscape of Molecular and Immunological Diagnostic Tests for Human Leishmaniasis: A Systematic Review and Meta-Analysis
Source: Pathogens. 2025 Nov 4;14(11):1123. doi: 10.3390/pathogens14111123 (PMC12655109; doi:10.3390/pathogens14111123)
Supplement: Supplementary file 1 [file pathogens-14-01123-s001.zip › pathogens-3910933-supplementary.pdf]

Supplementary Figures

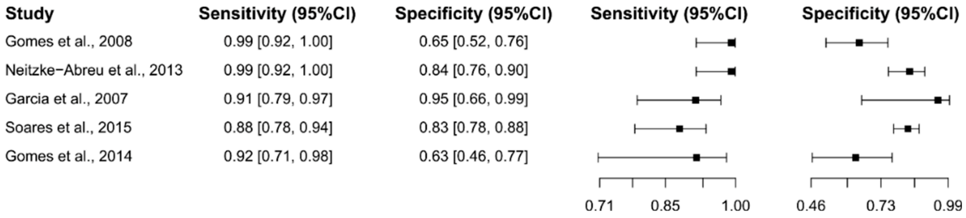

Figure S1. Study data and paired forest plot of the sensitivity and specificity of the leishmanin skin test (LST) in tegumentary leishmaniasis diagnosis. Data from each included study [46–50] are summarized. Sensitivity and specificity are reported with a mean (95% confidence limits). The forest plot depicts the estimated sensitivity and specificity (black squares) and their 95% confidence limits (horizontal black line).

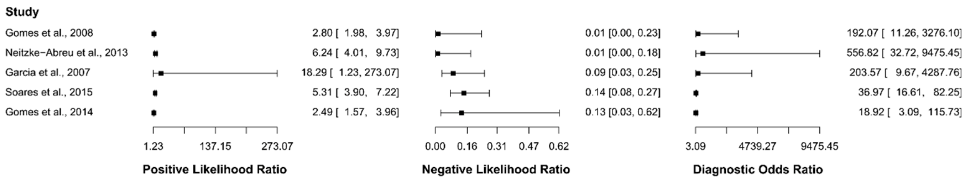

Figure S2. Study data and paired forest plot of the positive likelihood ratio, negative likelihood ratio, and diagnostic odds ratio of the leishmanin skin test (LST) in the diagnosis of tegumentary leishmaniasis. The positive likelihood ratio, negative likelihood ratio, and diagnostic odds ratio are reported with a mean (95% confidence limits) for the included studies [46–50].

Study	Sensitivity (95%CI)	Specificity (95%CI)
Carvalho et al., 2017	0.99 [0.92, 1.00]	0.97 [0.89, 0.99]
Rodrigues et al., 2019	0.98 [0.90, 0.99]	0.84 [0.72, 0.92]
Deepachandi et al., 2020	0.82 [0.76, 0.87]	0.99 [0.97, 1.00]
Ribeiro et al., 2018	0.99 [0.90, 1.00]	0.96 [0.86, 0.99]
Pedrosa et al., 2017	0.99 [0.90, 1.00]	0.97 [0.88, 0.99]
Massae et al., 2017	0.83 [0.73, 0.90]	0.83 [0.73, 0.90]
Massae et al., 2017	0.95 [0.87, 0.98]	0.92 [0.83, 0.96]
Massae et al., 2017	0.99 [0.93, 1.00]	0.98 [0.91, 0.99]
Massae et al., 2017	0.89 [0.79, 0.94]	0.95 [0.87, 0.98]
Bracamonteetal., 2020	0.98 [0.92, 0.99]	0.63 [0.49, 0.76]
Garcia et al., 1990	0.75 [0.65, 0.84]	0.99 [0.94, 1.00]
Menezes et al., 2014	0.98 [0.91, 0.99]	0.99 [0.91, 1.00]
Pedras et al., 2003	0.92 [0.73, 0.98]	0.70 [0.59, 0.79]
Pedras et al., 2003	0.97 [0.78, 1.00]	0.70 [0.59, 0.79]
Pedras et al., 2003	0.98 [0.81, 1.00]	0.70 [0.59, 0.79]
Pedras et al., 2003	0.78 [0.56, 0.90]	0.97 [0.91, 0.99]
Pedras et al., 2003	0.69 [0.46, 0.86]	0.97 [0.91, 0.99]
Pedras et al., 2003	0.69 [0.48, 0.85]	0.97 [0.91, 0.99]
Pedras et al., 2003	0.88 [0.67, 0.96]	0.83 [0.74, 0.90]
Pedras et al., 2003	0.97 [0.78, 1.00]	0.83 [0.74, 0.90]
Pedras et al., 2003	0.83 [0.63, 0.94]	0.83 [0.74, 0.90]
Szargiki et al., 2009	0.71 [0.59, 0.81]	0.82 [0.56, 0.94]
Szargiki et al., 2009	0.71 [0.59, 0.81]	0.31 [0.17, 0.48]
Szargiki et al., 2009	0.71 [0.59, 0.81]	0.14 [0.03, 0.43]
Szargiki et al., 2009	0.71 [0.59, 0.81]	0.83 [0.51, 0.96]
Szargiki et al., 2009	0.94 [0.85, 0.98]	0.89 [0.64, 0.97]
Szargiki et al., 2009	0.94 [0.85, 0.98]	0.56 [0.39, 0.72]
Szargiki et al., 2009	0.94 [0.85, 0.98]	0.23 [0.07, 0.52]
Szargiki et al., 2009	0.94 [0.85, 0.98]	0.50 [0.23, 0.77]
Salotra et al., 2003	0.94 [0.78, 0.99]	0.94 [0.78, 0.99]
Duarte et al., 2015	0.99 [0.90, 1.00]	0.98 [0.86, 1.00]
Duarte et al., 2015	0.99 [0.90, 1.00]	0.92 [0.77, 0.97]
Duarte et al., 2015	0.99 [0.90, 1.00]	0.85 [0.69, 0.94]
Duarte et al., 2015	0.99 [0.90, 1.00]	0.82 [0.66, 0.92]
Soares et al., 2015	0.90 [0.83, 0.95]	0.83 [0.78, 0.88]
Cataldo et al., 2010	0.89 [0.80, 0.94]	0.89 [0.80, 0.94]
Cataldo et al., 2010	0.89 [0.80, 0.94]	0.93 [0.85, 0.97]
Souza et al., 2013	0.65 [0.55, 0.73]	0.92 [0.84, 0.96]
Souza et al., 2013	0.82 [0.70, 0.90]	0.81 [0.72, 0.88]
Souza et al., 2013	0.73 [0.59, 0.83]	0.72 [0.62, 0.81]
Souza et al., 2013	0.66 [0.56, 0.74]	0.71 [0.61, 0.80]
Souza et al., 2013	0.60 [0.47, 0.72]	0.72 [0.62, 0.81]
Souza et al., 2013	0.71 [0.57, 0.82]	0.71 [0.61, 0.80]
Souza et al., 2013	0.41 [0.32, 0.51]	0.79 [0.70, 0.86]
Souza et al., 2013	0.86 [0.74, 0.93]	0.61 [0.51, 0.71]
Souza et al., 2013	0.77 [0.64, 0.86]	0.61 [0.51, 0.71]
Souza et al., 2013	0.40 [0.31, 0.50]	0.71 [0.61, 0.80]
Souza et al., 2013	0.44 [0.31, 0.57]	0.69 [0.59, 0.78]
Souza et al., 2013	0.51 [0.38, 0.64]	0.56 [0.45, 0.65]
Souza et al., 2013	0.53 [0.43, 0.62]	0.71 [0.61, 0.80]
Souza et al., 2013	0.81 [0.68, 0.89]	0.54 [0.44, 0.64]
Souza et al., 2013	0.61 [0.47, 0.73]	0.54 [0.44, 0.64]
Souza et al., 2013	0.71 [0.62, 0.79]	0.53 [0.43, 0.63]
Souza et al., 2013	0.73 [0.60, 0.83]	0.71 [0.61, 0.80]
Souza et al., 2013	0.55 [0.41, 0.68]	0.53 [0.43, 0.63]
Montoya et al., 1997	0.66 [0.54, 0.76]	0.86 [0.72, 0.94]
Montoya et al., 1997	0.75 [0.65, 0.84]	0.96 [0.85, 0.99]
Redhu et al., 2006	0.99 [0.91, 1.00]	1.00 [0.97, 1.00]
Mosleh et al., 1995	0.80 [0.65, 0.90]	0.97 [0.92, 0.99]
Zeyrek et al., 2007	0.92 [0.76, 0.97]	0.94 [0.83, 0.98]

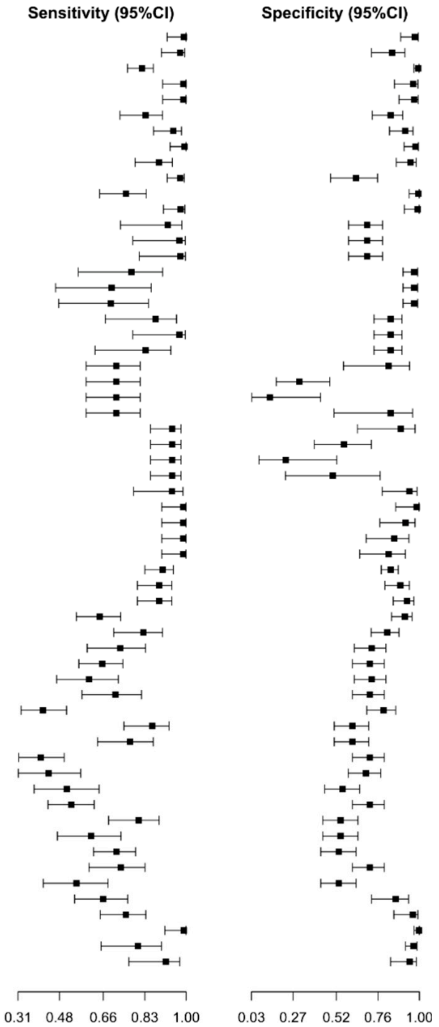

Figure S3. Study data and paired forest plot of the sensitivity and specificity of enzyme-linked immunosorbent assay (ELISA) in tegumentary leishmaniasis diagnosis. Data from each included study [47,51–69] are summarized. Sensitivity and specificity are reported with a mean (95% confidence limits). The forest plot depicts the estimated sensitivity and specificity (black squares) and their 95% confidence limits (horizontal black line).

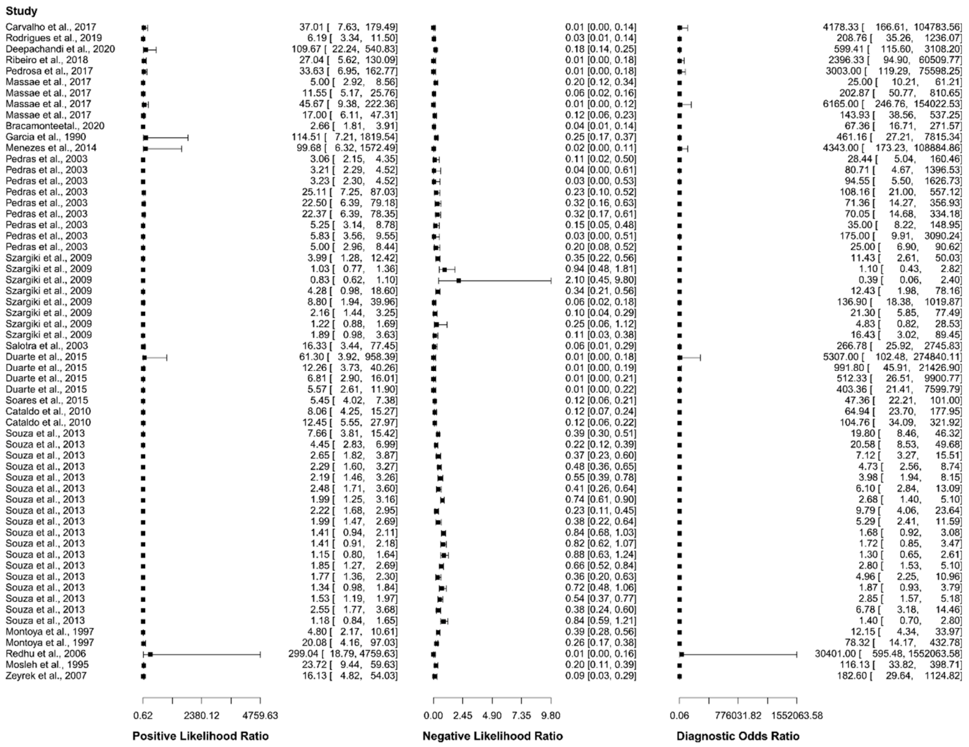

Figure S4. Study data and paired forest plot of the positive likelihood ratio, negative likelihood ratio, and diagnostic odds ratio of enzyme-linked immunosorbent assay (ELISA) in the diagnosis of tegumentary leishmaniasis. The positive likelihood ratio, negative likelihood ratio, and diagnostic odds ratio are reported with a mean (95% confidence limits) for the included studies [47,51–69].

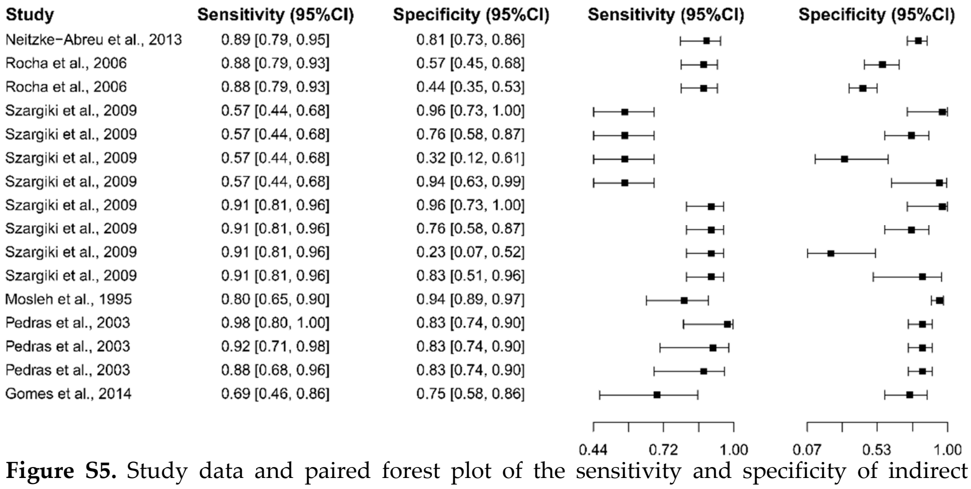

Figure S5. Study data and paired forest plot of the sensitivity and specificity of indirect fluorescent antibody test (IFAT) in tegumentary leishmaniasis diagnosis. Data from each included study [46,49,53,60,70,71] are summarized. Sensitivity and specificity are reported with a mean (95% confidence limits). The forest plot depicts the estimated sensitivity and specificity (black squares) and their 95% confidence limits (horizontal black line).

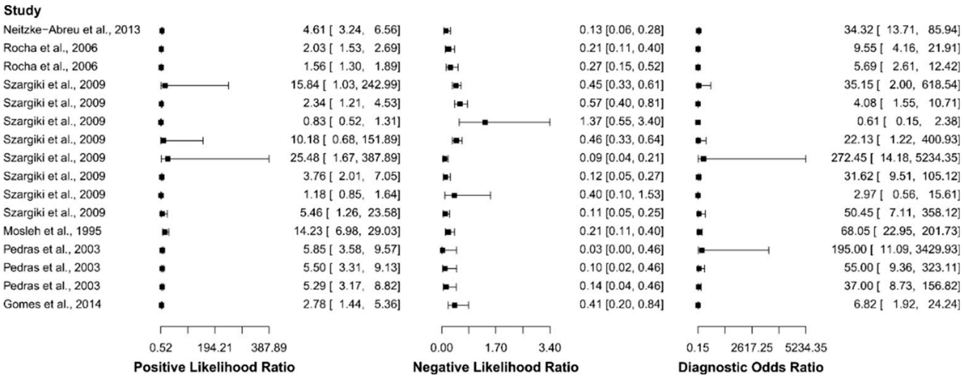

Figure S6. Study data and paired forest plot of the positive likelihood ratio, negative likelihood ratio, and diagnostic odds ratio of indirect fluorescent antibody test (IFAT) in the diagnosis of tegumentary leishmaniasis. The positive likelihood ratio, negative likelihood ratio, and diagnostic odds ratio are reported with a mean (95% confidence limits) for the included studies [46,49,53,60,70,71].

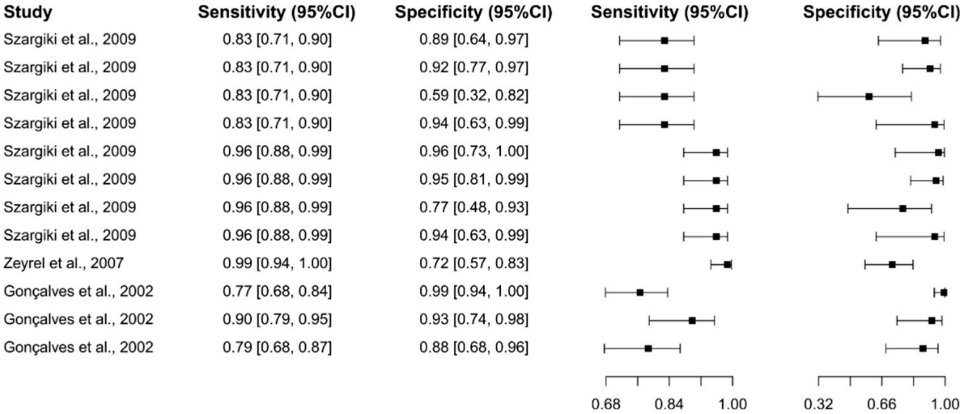

Figure S7. Study data and paired forest plot of the sensitivity and specificity of Western Blot (WB) in tegumentary leishmaniasis diagnosis. Data from each included study [53,61,72] are summarized. Sensitivity and specificity are reported with a mean (95% confidence limits). The forest plot depicts the estimated sensitivity and specificity (black squares) and their 95% confidence limits (horizontal black line).

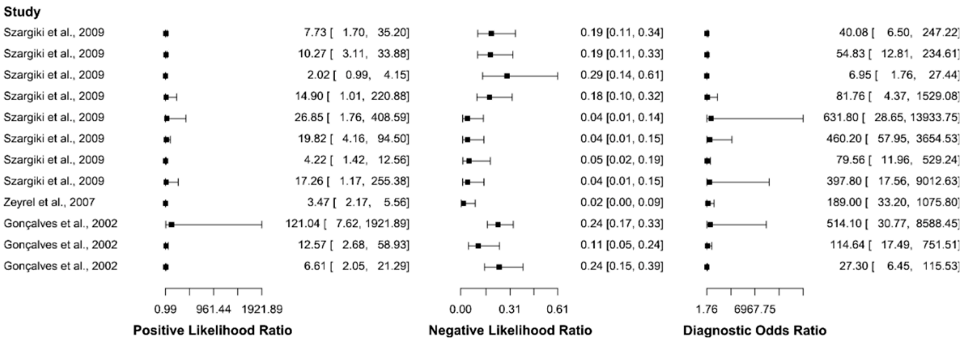

Figure S8. Study data and paired forest plot of the positive likelihood ratio, negative likelihood ratio, and diagnostic odds ratio of Western Blot (WB) in the diagnosis of tegumentary

leishmaniasis. The positive likelihood ratio, negative likelihood ratio, and diagnostic odds ratio are reported with a mean (95% confidence limits) for the included studies [53,61,72].

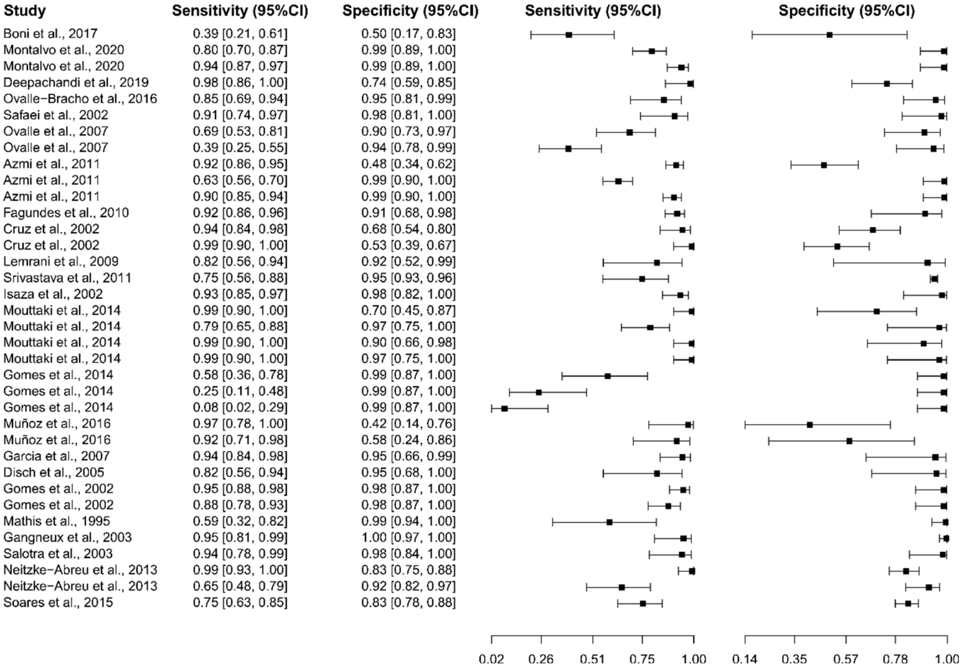

Figure S9. Study data and paired forest plot of the sensitivity and specificity of polymerase chain reaction (PCR) in tegumentary leishmaniasis diagnosis. Data from each included study [46–49,73–91] are summarized. Sensitivity and specificity are reported with a mean (95% confidence limits). The forest plot depicts the estimated sensitivity and specificity (black squares) and their 95% confidence limits (horizontal black line).

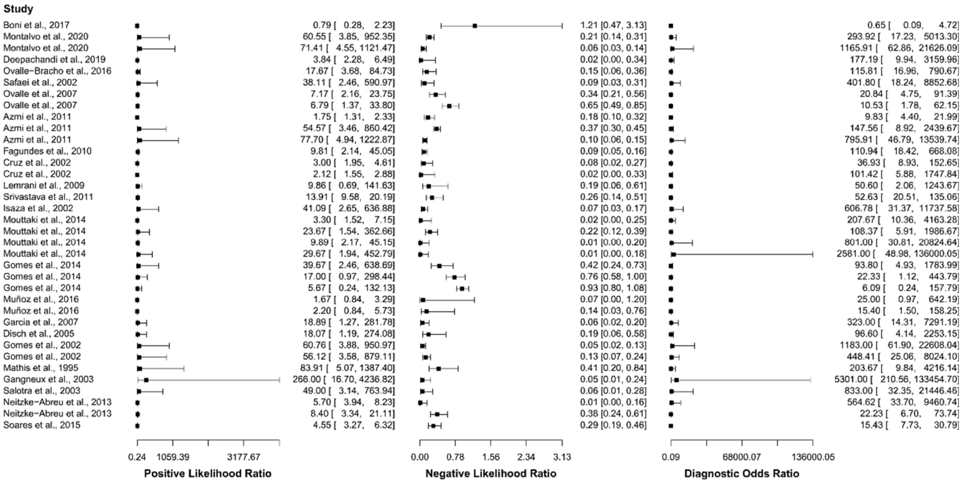

Figure S10. Study data and paired forest plot of the positive likelihood ratio, negative likelihood ratio, and diagnostic odds ratio of polymerase chain reaction (PCR) in the diagnosis of tegumentary leishmaniasis. The positive likelihood ratio, negative likelihood ratio, and

diagnostic odds ratio are reported with a mean (95% confidence limits) for the included studies [46–49,73–91].

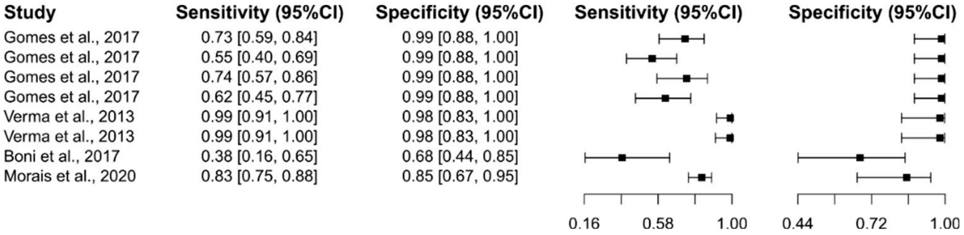

Figure S11. Study data and paired forest plot of the sensitivity and specificity of real-time polymerase chain reaction (qPCR) in tegumentary leishmaniasis diagnosis. Data from each included study [86,92–94] are summarized. Sensitivity and specificity are reported with a mean (95% confidence limits). The forest plot depicts the estimated sensitivity and specificity (black squares) and their 95% confidence limits (horizontal black line).

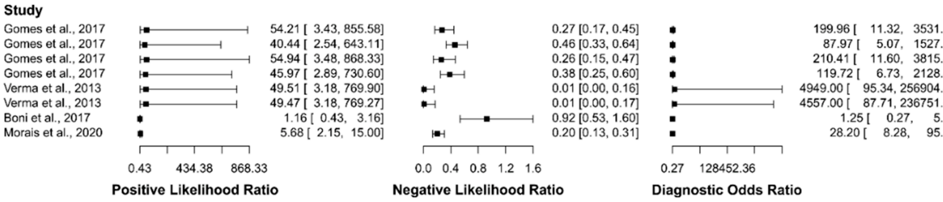

Figure S12. Study data and paired forest plot of the positive likelihood ratio, negative likelihood ratio, and diagnostic odds ratio of real-time polymerase chain reaction (qPCR) in the diagnosis of tegumentary leishmaniasis. The positive likelihood ratio, negative likelihood ratio, and diagnostic odds ratio are reported with a mean (95% confidence limits) for the included studies [86,92–94].

Study	Sensitivity (95%CI)	Specificity (95%CI)
Bangert et al., 2018	0.86 [0.80, 0.91]	0.86 [0.81, 0.90]
Vogt et al., 2018	0.84 [0.74, 0.90]	0.98 [0.94, 1.00]
Oliveira et al., 2017	0.99 [0.97, 1.00]	0.97 [0.91, 0.99]
Oliveira et al., 2017	0.96 [0.93, 0.98]	0.97 [0.91, 0.99]
Oliveira et al., 2017	0.98 [0.96, 0.99]	0.96 [0.89, 0.98]
Oliveira et al., 2017	0.98 [0.96, 0.99]	0.97 [0.91, 0.99]
Ben−Abid et al., 2017	0.51 [0.36, 0.67]	0.98 [0.90, 0.99]
Ben−Abid et al., 2017	0.79 [0.62, 0.90]	0.88 [0.78, 0.94]
Chappuis et al., 2006	0.84 [0.72, 0.91]	0.97 [0.90, 0.99]
Akhoundi et al., 2013	0.88 [0.75, 0.94]	0.93 [0.84, 0.97]
Akhoundi et al., 2013	0.88 [0.75, 0.94]	0.91 [0.82, 0.96]
Salam et al., 2011	0.74 [0.58, 0.86]	0.99 [0.89, 1.00]
Assis et al., 2011	0.90 [0.85, 0.93]	0.95 [0.90, 0.98]
Sundar et al., 2007	0.98 [0.96, 0.99]	0.94 [0.87, 0.97]
Sundar et al., 2007	0.98 [0.96, 0.99]	0.92 [0.84, 0.97]
Sundar et al., 2007	1.00 [0.98, 1.00]	0.94 [0.87, 0.97]
Sundar et al., 2007	1.00 [0.98, 1.00]	0.91 [0.82, 0.96]
Sundar et al., 2007	0.73 [0.67, 0.79]	0.99 [0.94, 1.00]
Sundar et al., 2007	0.73 [0.67, 0.79]	0.88 [0.78, 0.94]
Akhoundi et al., 2010	0.94 [0.60, 0.99]	0.87 [0.82, 0.91]
Oliveira et al., 2013	0.99 [0.94, 1.00]	0.97 [0.92, 0.99]
Oliveira et al., 2013	0.99 [0.94, 1.00]	1.00 [0.96, 1.00]
Lita et al., 2002	0.97 [0.88, 0.99]	0.98 [0.86, 1.00]
Osman et al., 2016	0.95 [0.89, 0.98]	0.43 [0.29, 0.58]
Osman et al., 2016	0.91 [0.84, 0.95]	0.43 [0.29, 0.58]
Oliveira et al., 2011	0.94 [0.87, 0.97]	0.99 [0.95, 1.00]
Oliveira et al., 2011	0.95 [0.88, 0.98]	1.00 [0.96, 1.00]
Oliveira et al., 2011	0.99 [0.95, 1.00]	0.97 [0.93, 0.99]
Hasnain et al., 2014	0.99 [0.91, 1.00]	0.99 [0.91, 1.00]
Hasnain et al., 2014	0.95 [0.85, 0.98]	0.99 [0.91, 1.00]
Bimal et al., 2005	0.91 [0.84, 0.95]	1.00 [0.99, 1.00]
Bimal et al., 2005	0.91 [0.84, 0.95]	1.00 [0.98, 1.00]
el Harith et al., 1995	0.99 [0.89, 1.00]	0.97 [0.79, 1.00]
Oliveira et al., 2009	0.93 [0.84, 0.97]	0.96 [0.91, 0.99]
Rijal et al., 2004	0.48 [0.40, 0.56]	0.50 [0.42, 0.57]
Abdallah et al., 2004	0.96 [0.88, 0.99]	0.95 [0.88, 0.98]
Abdallah et al., 2004	0.90 [0.79, 0.95]	0.96 [0.90, 0.98]
Singla et al., 1993	0.96 [0.87, 0.99]	0.99 [0.89, 1.00]
Oskman et al.,1999	0.92 [0.87, 0.95]	0.98 [0.92, 0.99]
Meredith et al., 1995	0.91 [0.80, 0.96]	1.00 [0.99, 1.00]
Moody et al., 1996	0.88 [0.77, 0.94]	0.95 [0.88, 0.98]
Schoone et al., 2001	0.90 [0.76, 0.96]	0.96 [0.72, 1.00]
Schoone et al., 2001	0.93 [0.79, 0.98]	0.96 [0.72, 1.00]
Schoone et al., 2001	0.91 [0.86, 0.94]	0.99 [0.90, 1.00]
Schoone et al., 2001	0.91 [0.86, 0.94]	0.92 [0.79, 0.97]
Lourenço et al., 2019	0.93 [0.86, 0.97]	0.97 [0.90, 0.99]
Lourenço et al., 2019	0.88 [0.75, 0.95]	0.89 [0.75, 0.95]
Pedras et al., 2008	0.96 [0.90, 0.99]	0.98 [0.93, 0.99]
Pedras et al., 2008	0.95 [0.88, 0.98]	0.88 [0.81, 0.93]
Machado de Assis et al., 2012	0.88 [0.84, 0.92]	0.95 [0.90, 0.98]
Abass et al., 2006	0.99 [0.89, 1.00]	0.50 [0.44, 0.55]
Abass et al., 2006	0.99 [0.89, 1.00]	0.99 [0.88, 1.00]
Bagchi et al., 1998	0.97 [0.88, 0.99]	0.87 [0.70, 0.95]
Bagchi et al., 1998	0.97 [0.88, 0.99]	0.98 [0.84, 1.00]
Bagchi et al., 1998	0.95 [0.86, 0.98]	0.94 [0.79, 0.99]
Bagchi et al., 1998	0.95 [0.86, 0.98]	0.98 [0.84, 1.00]
Kumar et al., 2006	0.93 [0.85, 0.97]	0.97 [0.95, 0.98]
Abass et al., 2013	0.94 [0.88, 0.97]	0.99 [0.94, 1.00]
Okong'o−Odera et al., 1993	0.94 [0.63, 0.99]	0.90 [0.76, 0.96]
Attar et al., 2001	0.78 [0.65, 0.86]	1.00 [0.98, 1.00]
Attar et al., 2001	0.78 [0.65, 0.86]	0.99 [0.92, 1.00]
Cañavate et al., 2011	0.88 [0.81, 0.93]	0.97 [0.93, 0.99]
Chappuis et al., 2003	0.90 [0.46, 0.99]	0.83 [0.51, 0.96]
Bern et al., 2000	0.97 [0.92, 0.99]	0.92 [0.86, 0.96]
Do Vale et al., 2020	0.86 [0.74, 0.93]	0.99 [0.92, 1.00]
Ayelign et al., 2020	0.97 [0.92, 0.99]	0.99 [0.94, 1.00]
Ayelign et al., 2020	0.99 [0.94, 1.00]	0.99 [0.94, 1.00]
Schallig et al., 2002	0.98 [0.82, 1.00]	0.98 [0.80, 1.00]
Silva et al., 2019	0.93 [0.83, 0.98]	0.97 [0.79, 1.00]
Silva et al., 2019	0.94 [0.86, 0.97]	0.97 [0.79, 1.00]
Silva et al., 2019	0.91 [0.83, 0.95]	0.97 [0.79, 1.00]
Sundar et al., 2006	0.96 [0.91, 0.98]	0.85 [0.81, 0.88]
Sundar et al., 2006	0.97 [0.93, 0.99]	0.87 [0.83, 0.90]
Sinha et al., 2008	0.99 [0.95, 1.00]	0.99 [0.91, 1.00]
Abass et al., 2015	0.93 [0.87, 0.97]	0.99 [0.94, 1.00]
Abass et al., 2015	0.92 [0.85, 0.95]	0.96 [0.89, 0.99]
Mansour et al., 2007	0.92 [0.86, 0.95]	1.00 [0.97, 1.00]

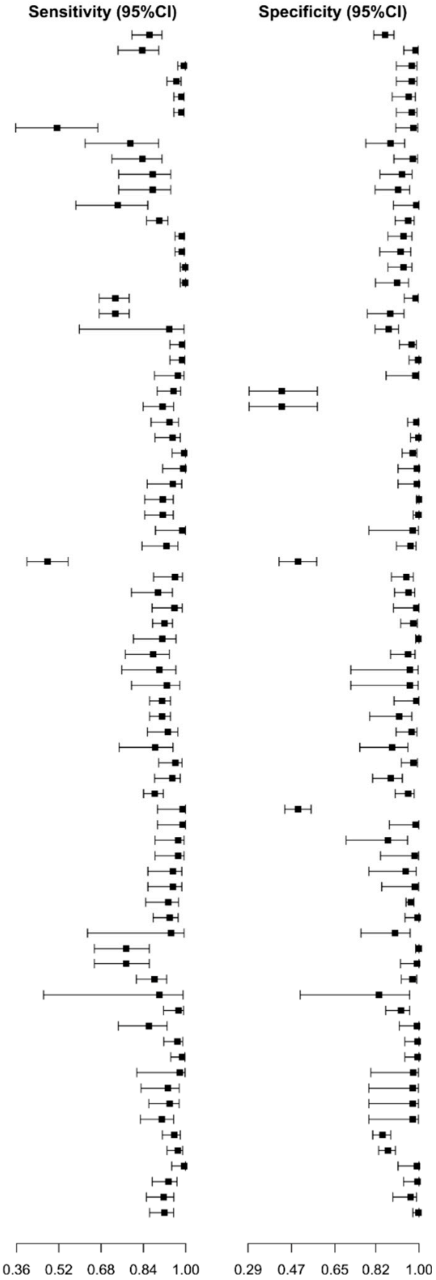

Figure S13. Study data and paired forest plot of the sensitivity and specificity of the direct agglutination test (DAT) in visceral leishmaniasis diagnosis. Data from each included study [52,98–141] are summarized. Sensitivity and specificity are reported with a mean (95% confidence limits). The forest plot depicts the estimated sensitivity and specificity (black squares) and their 95% confidence limits (horizontal black line).

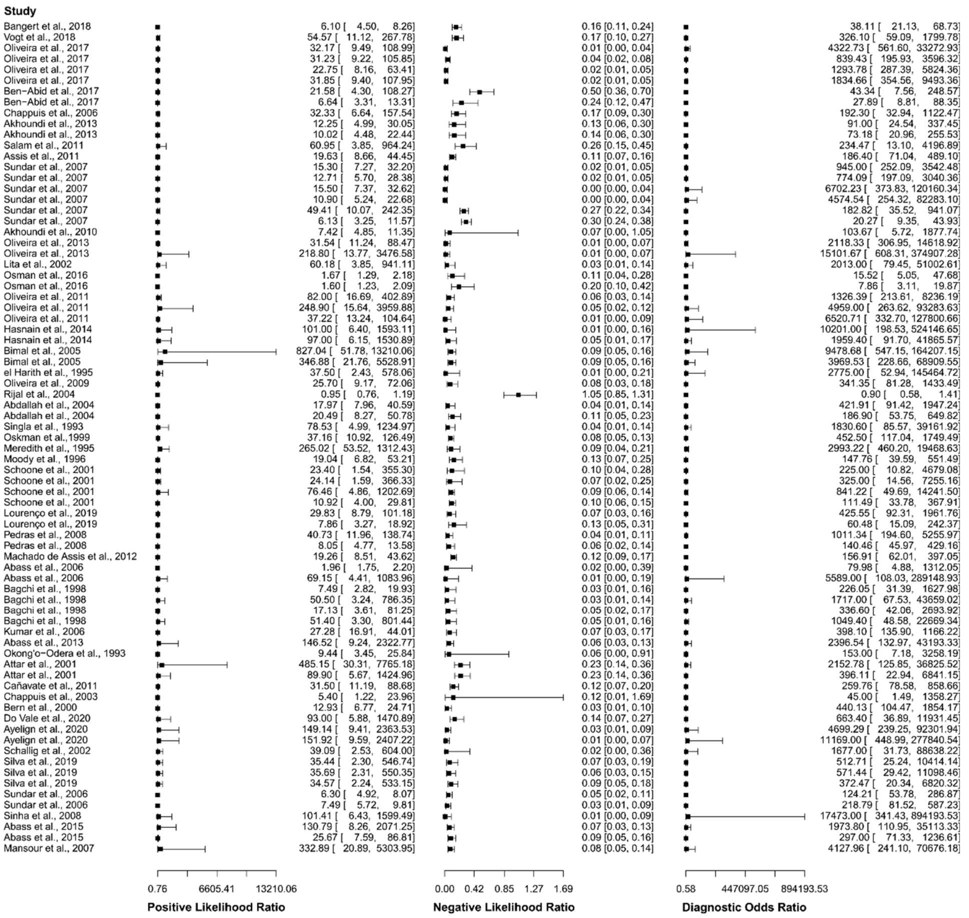

Figure S14. Study data and paired forest plot of the positive likelihood ratio, negative likelihood ratio, and diagnostic odds ratio of the direct agglutination test (DAT) in the diagnosis of visceral leishmaniasis. The positive likelihood ratio, negative likelihood ratio, and diagnostic odds ratio are reported with a mean (95% confidence limits) for the included studies [52,98–141].

Study	Sensitivity (95%CI)	Specificity (95%CI)
Hossain et al., 2021	0.97 [0.91, 0.99]	0.91 [0.82, 0.95]
Salles et al., 2016	0.98 [0.86, 1.00]	0.98 [0.85, 1.00]
Machado et al., 2020	0.99 [0.88, 1.00]	0.99 [0.94, 1.00]
Dias et al., 2018	0.99 [0.93, 1.00]	0.99 [0.92, 1.00]
Ribeiro et al., 2019	0.84 [0.74, 0.91]	0.72 [0.62, 0.80]
Ribeiro et al., 2019	0.87 [0.77, 0.93]	0.66 [0.57, 0.75]
Ribeiro et al., 2019	0.91 [0.82, 0.96]	0.87 [0.79, 0.92]
Abdus et al,. 2021	0.98 [0.92, 0.99]	0.97 [0.91, 0.99]
Abdus et al,. 2021	0.98 [0.92, 0.99]	0.94 [0.87, 0.97]
Saliba et al., 2019	0.96 [0.92, 0.99]	0.92 [0.87, 0.95]
Saliba et al., 2019	0.98 [0.94, 0.99]	0.92 [0.87, 0.95]
Costa et al., 2017	0.99 [0.89, 1.00]	0.96 [0.85, 0.99]
Costa et al., 2017	0.89 [0.75, 0.95]	0.89 [0.75, 0.95]
Costa et al., 2017	0.91 [0.79, 0.97]	0.96 [0.85, 0.99]
Costa et al., 2017	0.91 [0.79, 0.97]	0.84 [0.69, 0.92]
Oliveira−da−Silva et al., 2020	0.98 [0.84, 1.00]	0.94 [0.78, 0.99]
Lourenço et al., 2019	0.77 [0.67, 0.85]	0.93 [0.85, 0.97]
Lourenço et al., 2019	0.63 [0.47, 0.76]	0.96 [0.85, 0.99]
Lourenço et al., 2019	0.93 [0.86, 0.97]	0.77 [0.67, 0.85]
Lourenço et al., 2019	0.78 [0.63, 0.88]	0.89 [0.75, 0.95]
Lourenço et al., 2019	0.86 [0.77, 0.92]	0.96 [0.89, 0.98]
Lourenço et al., 2019	0.65 [0.50, 0.78]	0.94 [0.82, 0.98]
Abeijon et al., 2020	0.90 [0.72, 0.97]	0.95 [0.68, 1.00]
Abeijon et al., 2019	0.99 [0.89, 1.00]	0.74 [0.57, 0.86]
Braz et al., 2002	0.93 [0.87, 0.96]	0.97 [0.90, 0.99]
Daprà et al., 2008	0.91 [0.88, 0.94]	0.97 [0.96, 0.98]
Vallur et al., 2015	0.93 [0.81, 0.97]	0.95 [0.68, 1.00]
Vallur et al., 2015	0.96 [0.88, 0.99]	0.95 [0.68, 1.00]
Vallur et al., 2015	0.96 [0.73, 1.00]	0.95 [0.68, 1.00]
Vallur et al., 2015	0.88 [0.75, 0.94]	0.95 [0.68, 1.00]
Vaish et al., 2011	0.99 [0.97, 1.00]	0.92 [0.85, 0.96]
Vaish et al., 2011	0.99 [0.97, 1.00]	0.90 [0.83, 0.94]
Ghosh et al., 2016	0.98 [0.93, 1.00]	0.96 [0.89, 0.98]
Ghosh et al., 2016	0.97 [0.91, 0.99]	0.97 [0.91, 0.99]
Ghosh et al., 2016	0.95 [0.88, 0.98]	0.97 [0.91, 0.99]
Pedras et al., 2008	0.88 [0.80, 0.93]	0.92 [0.85, 0.96]
Pedras et al., 2008	0.89 [0.81, 0.94]	0.81 [0.72, 0.87]
Vaish et al., 2012	0.83 [0.75, 0.89]	0.99 [0.91, 1.00]
Vaish et al., 2012	0.83 [0.75, 0.89]	0.90 [0.83, 0.95]
Vaish et al., 2012	0.83 [0.75, 0.89]	0.88 [0.75, 0.94]
Vaish et al., 2012	1.00 [0.96, 1.00]	0.97 [0.88, 0.99]
Vaish et al., 2012	1.00 [0.96, 1.00]	0.88 [0.80, 0.93]
Vaish et al., 2012	1.00 [0.96, 1.00]	0.88 [0.75, 0.94]
Machado de Assis et al., 2012	0.99 [0.97, 1.00]	0.82 [0.74, 0.88]
Abass et al., 2006	0.96 [0.86, 0.99]	0.99 [0.88, 1.00]
Abass et al., 2006	0.96 [0.86, 0.99]	1.00 [0.97, 1.00]
De Doncker et al., 2005	0.83 [0.72, 0.91]	0.86 [0.72, 0.94]
Costa et al., 2012	0.95 [0.80, 0.99]	0.79 [0.56, 0.92]
Costa et al., 2012	0.98 [0.85, 1.00]	0.91 [0.69, 0.98]
Costa et al., 2012	0.81 [0.64, 0.91]	0.79 [0.56, 0.92]
Costa et al., 2012	0.98 [0.85, 1.00]	0.91 [0.69, 0.98]
Costa et al., 2012	0.91 [0.76, 0.97]	0.91 [0.69, 0.98]
Costa et al., 2012	0.98 [0.85, 1.00]	0.91 [0.69, 0.98]
Costa et al., 2012	0.98 [0.85, 1.00]	0.97 [0.77, 1.00]
Costa et al., 2012	0.98 [0.85, 1.00]	0.91 [0.69, 0.98]
Costa et al., 2012	0.71 [0.53, 0.84]	0.97 [0.77, 1.00]
Costa et al., 2012	0.95 [0.80, 0.99]	0.91 [0.69, 0.98]
Maache et al., 2005	0.98 [0.81, 1.00]	0.86 [0.57, 0.97]
Abeijon et al., 2013	0.88 [0.68, 0.96]	0.99 [0.90, 1.00]
Kumar et al., 2006	0.90 [0.81, 0.95]	0.98 [0.96, 0.99]
Kumar et al., 2006	0.96 [0.89, 0.99]	0.98 [0.97, 0.99]
Fonseca et al., 2014	0.66 [0.55, 0.75]	0.59 [0.32, 0.82]
Fonseca et al., 2014	0.57 [0.47, 0.68]	0.50 [0.25, 0.75]
Fonseca et al., 2014	0.67 [0.56, 0.77]	0.77 [0.48, 0.93]
Zeyrek et al., 2007	0.78 [0.69, 0.84]	0.94 [0.83, 0.98]
Bandyopadhyay et al., 2004	0.91 [0.78, 0.97]	0.98 [0.81, 1.00]
Abass et al., 2013	0.98 [0.93, 0.99]	0.96 [0.88, 0.98]
Abass et al., 2013	0.96 [0.90, 0.98]	0.94 [0.87, 0.98]
Mohapatra et al., 2010	0.78 [0.65, 0.87]	0.83 [0.64, 0.93]
Mohapatra et al., 2010	0.38 [0.27, 0.51]	0.79 [0.60, 0.90]
Mohapatra et al., 2010	0.99 [0.92, 1.00]	0.94 [0.78, 0.99]
Galaï et al., 2011	0.99 [0.89, 1.00]	0.94 [0.82, 0.98]
Galaï et al., 2011	0.99 [0.89, 1.00]	0.96 [0.86, 0.99]
Barbosa−De−Deus et al., 2002	0.91 [0.80, 0.96]	1.00 [0.97, 1.00]
Islam et al., 2002	0.94 [0.85, 0.98]	0.99 [0.93, 1.00]
Islam et al., 2002	0.94 [0.85, 0.98]	0.99 [0.92, 1.00]
Islam et al., 2002	0.93 [0.84, 0.97]	0.99 [0.93, 1.00]
Islam et al., 2002	0.93 [0.84, 0.97]	0.99 [0.92, 1.00]
Islam et al., 2002	0.66 [0.52, 0.78]	0.89 [0.64, 0.97]
Islam et al., 2002	0.66 [0.52, 0.78]	0.97 [0.75, 1.00]
Islam et al., 2002	0.38 [0.25, 0.52]	0.96 [0.73, 1.00]
Islam et al., 2002	0.38 [0.25, 0.52]	0.97 [0.75, 1.00]
Islam et al., 2002	0.89 [0.76, 0.95]	0.99 [0.92, 1.00]
Islam et al., 2002	0.89 [0.76, 0.95]	0.99 [0.92, 1.00]
Kumar et al., 2011	0.88 [0.80, 0.93]	0.99 [0.95, 1.00]
Kumar et al., 2011	0.88 [0.80, 0.93]	0.96 [0.90, 0.98]
Kumar et al., 2011	0.88 [0.80, 0.93]	0.97 [0.92, 0.99]
Celeste et al., 2014	0.99 [0.92, 1.00]	0.98 [0.86, 1.00]
Celeste et al., 2014	0.99 [0.92, 1.00]	0.97 [0.90, 0.99]
Celeste et al., 2014	0.87 [0.76, 0.93]	0.98 [0.86, 1.00]
Celeste et al., 2014	0.87 [0.76, 0.93]	0.87 [0.78, 0.93]
Moreno et al., 2006	0.32 [0.24, 0.42]	0.71 [0.61, 0.79]
Moreno et al., 2006	0.34 [0.26, 0.44]	0.76 [0.67, 0.84]
Lakhal et al., 2012	0.97 [0.86, 0.99]	0.99 [0.94, 1.00]
Lakhal et al., 2012	0.97 [0.86, 0.99]	0.96 [0.89, 0.99]
Mansour et al., 2007	0.93 [0.87, 0.96]	0.95 [0.91, 0.98]
Kumar et al., 2012	0.95 [0.89, 0.98]	0.99 [0.94, 1.00]
Kumar et al., 2012	0.95 [0.89, 0.98]	0.96 [0.90, 0.99]
Kumar et al., 2012	0.95 [0.89, 0.98]	0.97 [0.91, 0.99]
Oliveira et al., 2011	0.72 [0.56, 0.84]	0.93 [0.87, 0.97]
Oliveira et al., 2011	0.96 [0.84, 0.99]	0.97 [0.92, 0.99]
Oliveira et al., 2011	0.37 [0.20, 0.57]	0.96 [0.91, 0.98]
Oliveira et al., 2011	0.80 [0.60, 0.92]	0.94 [0.88, 0.97]
Oliveira et al., 2011	0.85 [0.65, 0.94]	0.90 [0.83, 0.94]
Kurkjian., 2005	0.96 [0.83, 0.99]	0.99 [0.99, 0.99]
Fargeas et al., 1996	0.79 [0.63, 0.89]	0.78 [0.67, 0.86]
Silva et al., 2011	0.68 [0.59, 0.75]	0.78 [0.70, 0.84]
Silva et al., 2011	0.59 [0.50, 0.67]	0.73 [0.65, 0.80]
Silva et al., 2011	0.82 [0.74, 0.88]	0.27 [0.20, 0.35]
Kumar et al., 2011	0.94 [0.85, 0.97]	0.97 [0.88, 0.99]
Kumar et al., 2011	0.94 [0.85, 0.97]	0.99 [0.93, 1.00]
Kumar et al., 2011	0.94 [0.85, 0.97]	0.97 [0.86, 0.99]
De Assis et al., 2009	0.95 [0.86, 0.98]	0.93 [0.79, 0.98]
De Assis et al., 2009	0.87 [0.77, 0.93]	0.84 [0.69, 0.93]
Lemos et al,. 2003	0.90 [0.83, 0.94]	0.95 [0.68, 1.00]
Garcia et al., 2009	0.96 [0.84, 0.99]	0.91 [0.80, 0.96]
Vallur et al., 2016	0.33 [0.25, 0.42]	0.93 [0.82, 0.97]
Vallur et al., 2016	0.63 [0.54, 0.72]	0.89 [0.77, 0.95]
Vallur et al., 2016	0.79 [0.70, 0.85]	0.87 [0.75, 0.94]
Silva et al., 2019	0.86 [0.74, 0.93]	0.66 [0.43, 0.83]
Silva et al., 2019	0.87 [0.78, 0.93]	0.66 [0.43, 0.83]
Silva et al., 2019	0.78 [0.68, 0.85]	0.66 [0.43, 0.83]
Abass et al., 2015	0.97 [0.92, 0.99]	0.99 [0.94, 1.00]
Abass et al., 2015	0.96 [0.91, 0.98]	0.95 [0.87, 0.98]
Abass et al., 2015	0.95 [0.90, 0.98]	0.99 [0.94, 1.00]
Abass et al., 2015	0.96 [0.91, 0.98]	0.96 [0.89, 0.99]
Chauhan et al., 2016	0.99 [0.91, 1.00]	0.98 [0.81, 1.00]
Chauhan et al., 2016	0.95 [0.85, 0.98]	0.98 [0.81, 1.00]
Siqueira et al., 2021	0.97 [0.88, 0.99]	0.98 [0.82, 1.00]
Siqueira et al., 2021	0.97 [0.88, 0.99]	0.93 [0.76, 0.98]
Siqueira et al., 2021	0.97 [0.88, 0.99]	0.72 [0.51, 0.86]
Siqueira et al., 2021	0.81 [0.69, 0.90]	0.80 [0.60, 0.92]
Figueiredo et al., 2021	0.97 [0.91, 0.99]	1.00 [0.96, 1.00]
Figueiredo et al., 2021	0.82 [0.71, 0.90]	1.00 [0.96, 1.00]
Lévêque et al., 2020	0.89 [0.84, 0.93]	0.96 [0.91, 0.98]
Lévêque et al., 2020	0.93 [0.88, 0.96]	1.00 [0.97, 1.00]
Lévêque et al., 2020	0.80 [0.74, 0.86]	0.99 [0.95, 1.00]
Lévêque et al., 2020	0.94 [0.89, 0.96]	0.99 [0.95, 1.00]
Bagchi et al., 1998	0.93 [0.83, 0.98]	0.91 [0.74, 0.97]
Bagchi et al., 1998	0.93 [0.83, 0.98]	0.98 [0.84, 1.00]
Okong'o−Odera et al., 1993	0.94 [0.63, 0.99]	0.96 [0.83, 0.99]
Okong'o−Odera et al., 1993	0.39 [0.15, 0.69]	0.99 [0.88, 1.00]
Menezes et al., 2014	0.97 [0.89, 0.99]	0.95 [0.85, 0.98]

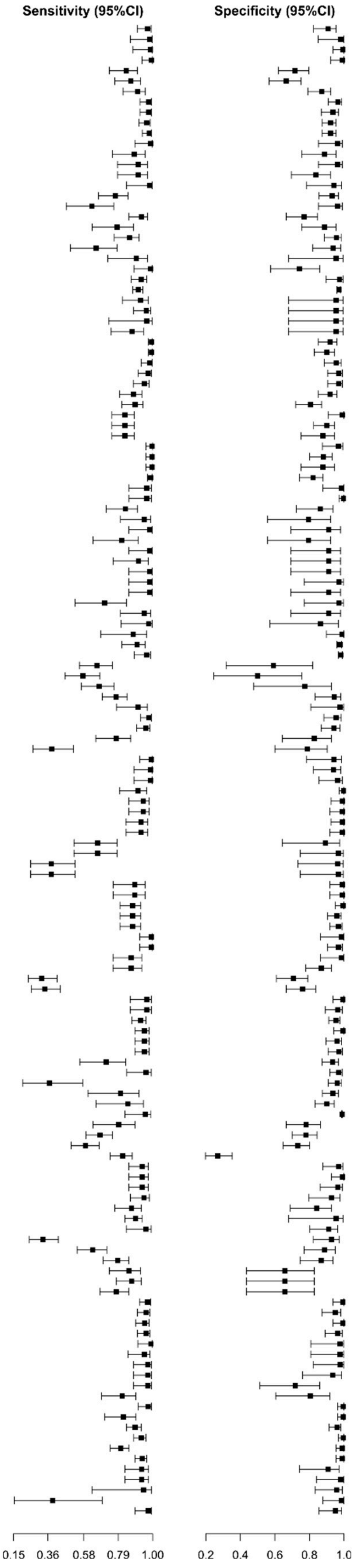

Figure S15. Study data and paired forest plot of the sensitivity and specificity of enzyme-linked immunosorbent assay (ELISA) in visceral leishmaniasis diagnosis. Data from each included study [52,61,69,116–119,121–123,132,135,136,142–186] are summarized. Sensitivity and specificity are reported with a mean (95% confidence limits). The forest plot depicts the estimated sensitivity and specificity (black squares) and their 95% confidence limits (horizontal black line).

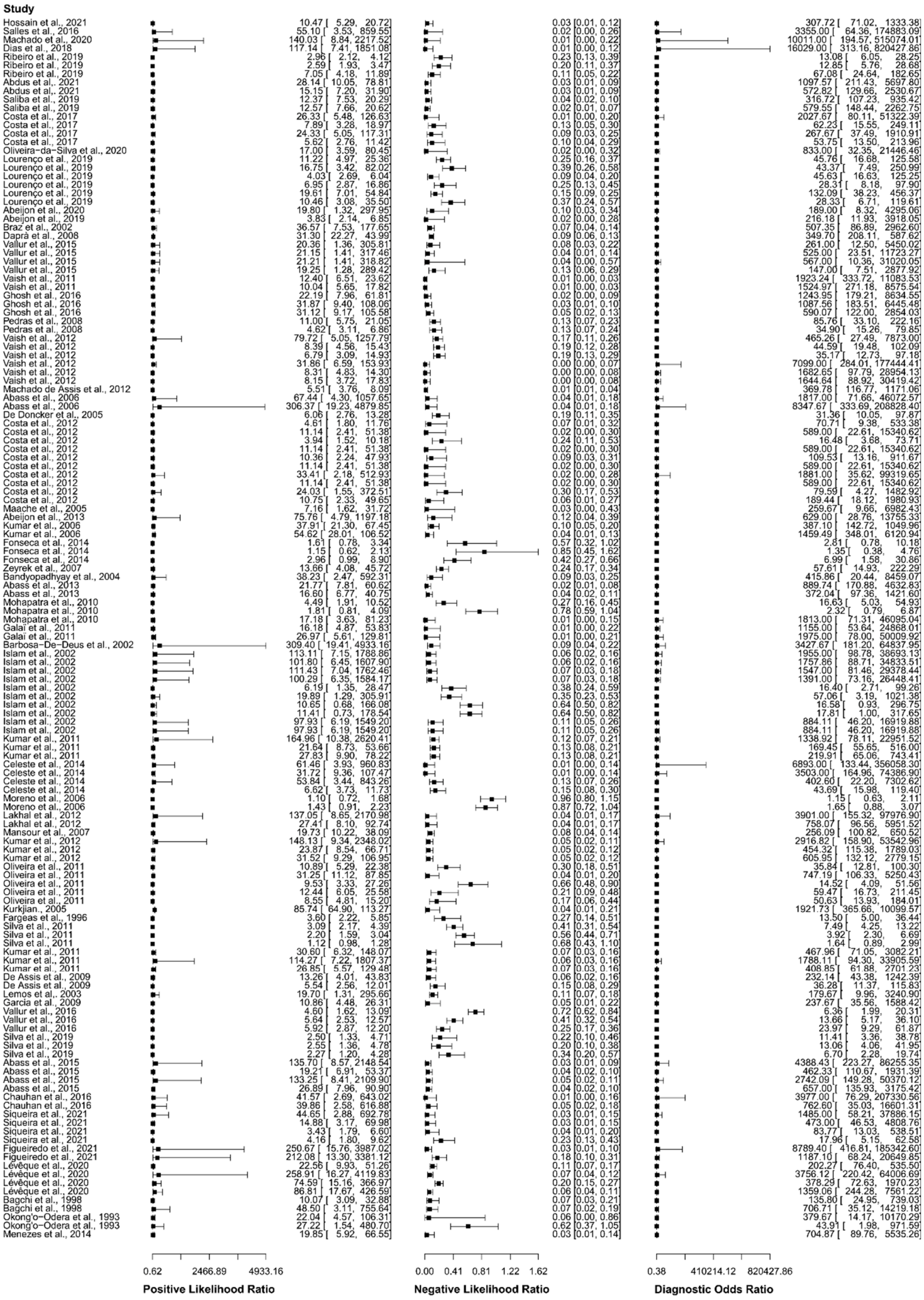

Figure S16. Study data and paired forest plot of the positive likelihood ratio, negative likelihood ratio, and diagnostic odds ratio of enzyme-linked immunosorbent assay (ELISA) in the diagnosis of visceral leishmaniasis. The positive likelihood ratio, negative likelihood ratio,

and diagnostic odds ratio are reported with a mean (95% confidence limits) for the included studies [52,61,69,116–119,121–123,132,135,136,142–186].

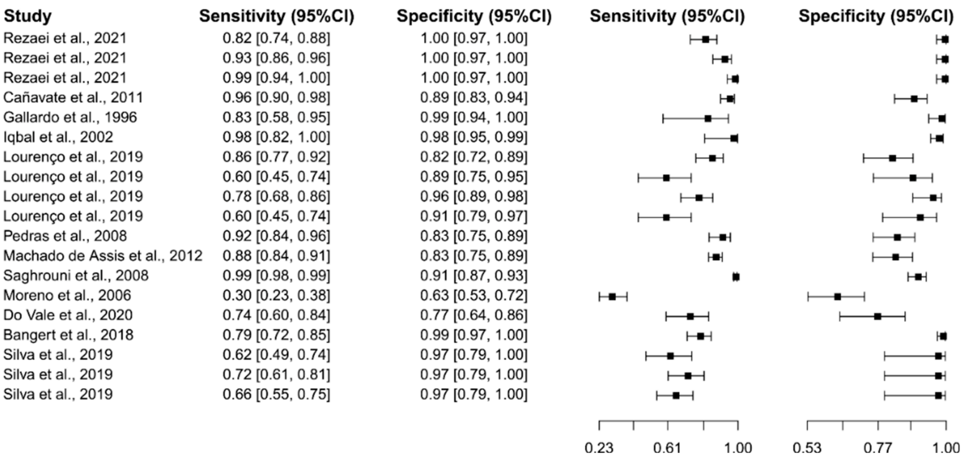

Figure S17. Study data and paired forest plot of the sensitivity and specificity of indirect fluorescent antibody test (IFAT) in visceral leishmaniasis diagnosis. Data from each included study [52,98,116,117,125,128,132,171,187–190] are summarized. Sensitivity and specificity are reported with a mean (95% confidence limits). The forest plot depicts the estimated sensitivity and specificity (black squares) and their 95% confidence limits (horizontal black line).

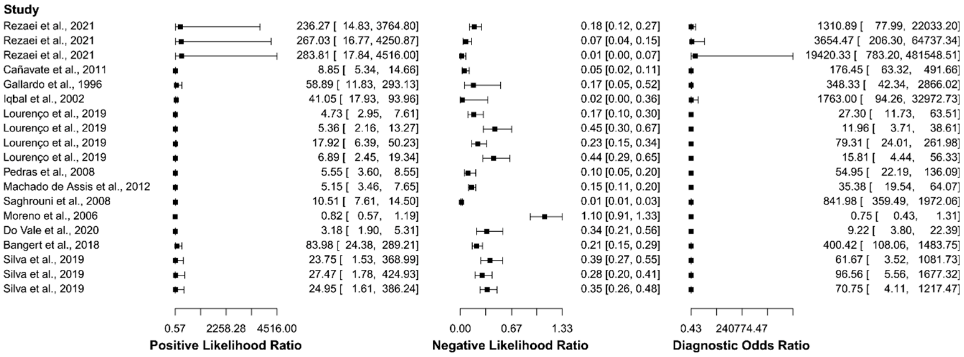

Figure S18. Study data and paired forest plots of the positive likelihood ratio, negative likelihood ratio, and diagnostic odds ratio of indirect fluorescent antibody test (IFAT) in the diagnosis of visceral leishmaniasis. The positive likelihood ratio, negative likelihood ratio, and diagnostic odds ratio are reported with a mean (95% confidence limits) for the included studies [52,98,116,117,125,128,132,171,187–190].

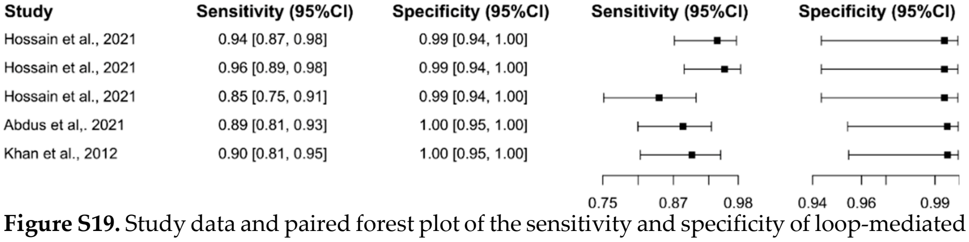

Figure S19. Study data and paired forest plot of the sensitivity and specificity of loop-mediated

isothermal amplification (LAMP) in visceral leishmaniasis diagnosis. Data from each included study [142,193,196] are summarized. Sensitivity and specificity are reported with a mean (95% confidence limits). The forest plot depicts the estimated sensitivity and specificity (black squares) and their 95% confidence limits (horizontal black line).

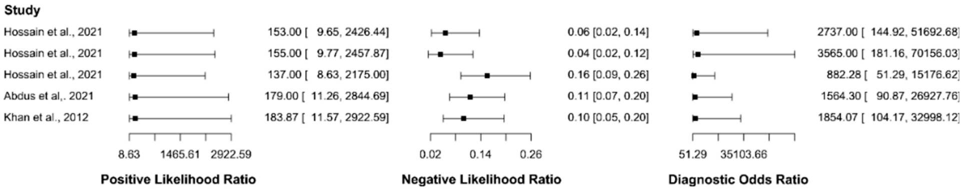

Figure S20. Study data and paired forest plot of the positive likelihood ratio, negative likelihood ratio, and diagnostic odds ratio of loop-mediated isothermal amplification (LAMP) in the diagnosis of visceral leishmaniasis. The positive likelihood ratio, negative likelihood ratio, and diagnostic odds ratio are reported with a mean (95% confidence limits) for the included studies [142,193,196].

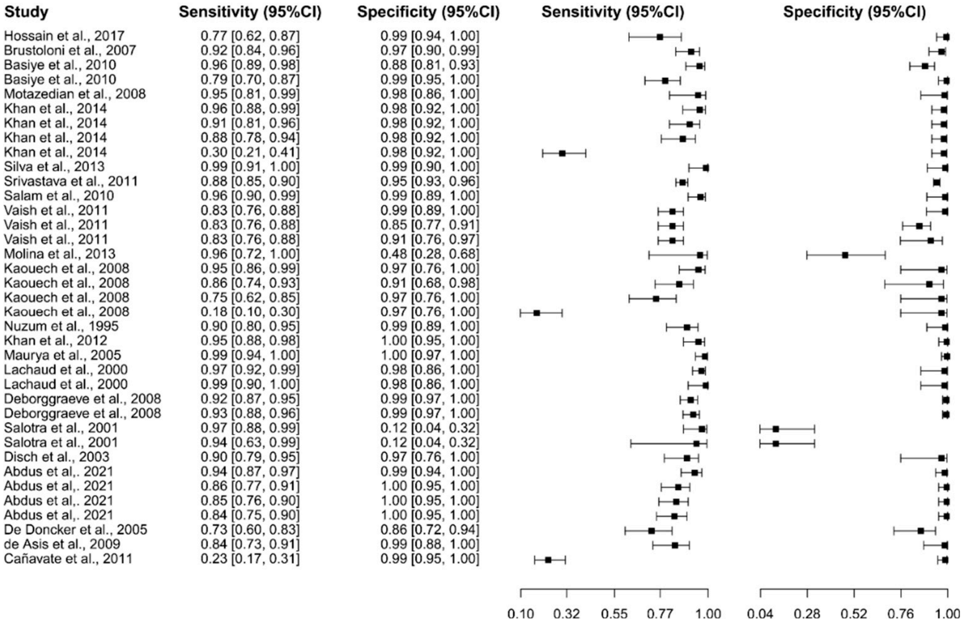

Figure S21. Study data and paired forest plot of the sensitivity and specificity of polymerase chain reaction (PCR) in visceral leishmaniasis diagnosis. Data from each included study [77,85,125,159,179,193,196–211] are summarized. Sensitivity and specificity are reported with a mean (95% confidence limits). The forest plot depicts the estimated sensitivity and specificity (black squares) and their 95% confidence limits (horizontal black line).

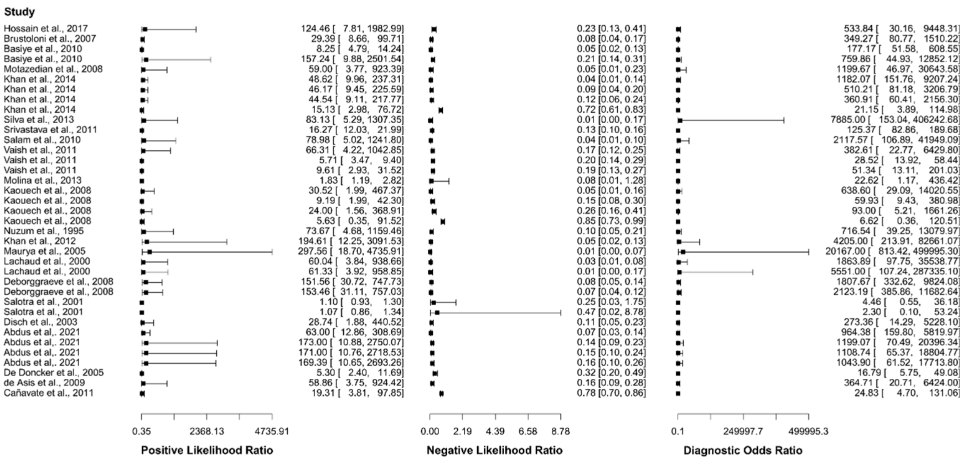

Figure S22. Study data and paired forest plot of the positive likelihood ratio, negative likelihood ratio, and diagnostic odds ratio of polymerase chain reaction (PCR) in the diagnosis of visceral leishmaniasis. The positive likelihood ratio, negative likelihood ratio, and diagnostic odds ratio are reported with a mean (95% confidence limits) for the included studies [77,85,125,159,179,193,196–211].

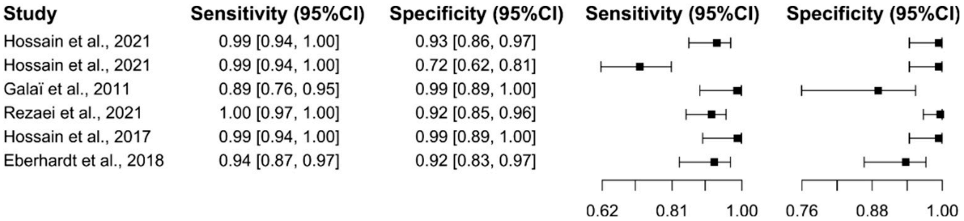

Figure S23. Study data and paired forest plot of the sensitivity and specificity of real-time polymerase chain reaction (qPCR) in visceral leishmaniasis diagnosis. Data from each included study [142,166,189,210,212] are summarized. Sensitivity and specificity are reported with a mean (95% confidence limits). The forest plot depicts the estimated sensitivity and specificity (black squares) and their 95% confidence limits (horizontal black line).

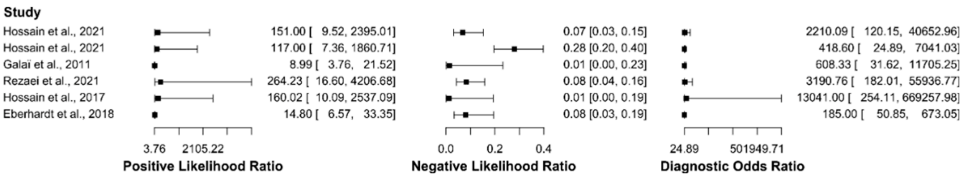

Figure S24. Study data and paired forest plot of the positive likelihood ratio, negative likelihood ratio, and diagnostic odds ratio of real-time polymerase chain reaction (qPCR) in the diagnosis of visceral leishmaniasis. The positive likelihood ratio, negative likelihood ratio, and diagnostic odds ratio are reported with a mean (95% confidence limits) for the included studies [142,166,189,210,212].

Study	Sensitivity (95%CI)	Specificity (95%CI)
Do Vale et al., 2020	0.81 [0.68, 0.89]	0.95 [0.86, 0.99]
Bangert et al., 2018	0.78 [0.70, 0.84]	1.00 [0.98, 1.00]
Siqueira et al., 2021	0.88 [0.72, 0.96]	0.99 [0.89, 1.00]
Siqueira et al., 2021	0.78 [0.61, 0.89]	0.96 [0.86, 0.99]
Schallig et al., 2002	0.84 [0.64, 0.94]	0.82 [0.61, 0.93]
Silva et al., 2019	0.91 [0.81, 0.96]	0.97 [0.79, 1.00]
Silva et al., 2019	0.95 [0.88, 0.98]	0.97 [0.79, 1.00]
Silva et al., 2019	0.87 [0.79, 0.93]	0.97 [0.79, 1.00]
Sundar et al., 2006	0.99 [0.96, 1.00]	0.89 [0.86, 0.92]
Moura et al., 2013	0.72 [0.64, 0.79]	0.99 [0.97, 1.00]
Sinha et al., 2008	0.98 [0.93, 1.00]	0.99 [0.91, 1.00]
Abass et al., 2015	0.88 [0.81, 0.93]	0.96 [0.89, 0.99]
Abass et al., 2015	0.96 [0.91, 0.98]	0.96 [0.89, 0.99]
Abass et al., 2015	0.70 [0.60, 0.78]	0.99 [0.94, 1.00]
Abass et al., 2015	0.96 [0.91, 0.98]	0.96 [0.89, 0.99]
Singh et al., 2013	1.00 [0.99, 1.00]	0.97 [0.94, 0.98]
Singh et al., 2013	0.96 [0.94, 0.98]	1.00 [0.99, 1.00]
Abdus et al,. 2021	0.99 [0.94, 1.00]	0.96 [0.90, 0.98]
Abdus et al,. 2021	0.99 [0.94, 1.00]	0.90 [0.82, 0.94]
Figueiredo et al., 2021	0.91 [0.84, 0.96]	0.97 [0.91, 0.99]
Figueiredo et al., 2021	0.73 [0.59, 0.84]	0.90 [0.80, 0.95]
Lévêque et al., 2020	0.85 [0.79, 0.89]	0.99 [0.95, 1.00]
Lévêque et al., 2020	0.90 [0.85, 0.93]	0.95 [0.90, 0.98]
Lourenço et al., 2019	0.96 [0.89, 0.98]	0.96 [0.89, 0.98]
Lourenço et al., 2019	0.63 [0.47, 0.76]	0.96 [0.85, 0.99]
Lourenço et al., 2019	0.92 [0.84, 0.96]	0.94 [0.87, 0.98]
Lourenço et al., 2019	0.47 [0.33, 0.63]	0.96 [0.85, 0.99]
Vaish et al., 2012	0.82 [0.74, 0.88]	0.91 [0.79, 0.96]
Vaish et al., 2012	0.82 [0.74, 0.88]	0.91 [0.84, 0.95]
Vaish et al., 2012	0.82 [0.74, 0.88]	0.83 [0.70, 0.91]
Vaish et al., 2012	1.00 [0.96, 1.00]	0.99 [0.91, 1.00]
Vaish et al., 2012	1.00 [0.96, 1.00]	0.94 [0.88, 0.97]
Vaish et al., 2012	1.00 [0.96, 1.00]	0.94 [0.84, 0.98]
Machado de Assis et al., 2012	0.93 [0.89, 0.95]	1.00 [0.96, 1.00]
Abass et al., 2013	0.81 [0.72, 0.87]	0.98 [0.92, 1.00]
Saghrouni et al., 2008	0.87 [0.84, 0.90]	0.94 [0.91, 0.96]
Lemos et al,. 2003	0.90 [0.83, 0.94]	0.95 [0.68, 1.00]
Herrera et al., 2019	0.91 [0.83, 0.95]	0.89 [0.80, 0.94]
Herrera et al., 2019	0.91 [0.83, 0.95]	0.93 [0.84, 0.97]
Cañavate et al., 2011	0.90 [0.83, 0.94]	0.90 [0.83, 0.94]
Cañavate et al., 2011	0.87 [0.79, 0.92]	0.91 [0.84, 0.95]
Iqbal et al., 2002	0.78 [0.54, 0.92]	0.99 [0.92, 1.00]
Berna et al., 2000	0.93 [0.86, 0.97]	1.00 [0.96, 1.00]
Ayelign et al., 2020	0.96 [0.90, 0.98]	0.99 [0.94, 1.00]
Chappuis et al., 2006	0.89 [0.81, 0.94]	0.89 [0.78, 0.95]
Assis et al., 2011	0.93 [0.88, 0.96]	0.96 [0.91, 0.98]
Osman et al., 2016	0.76 [0.66, 0.83]	0.41 [0.27, 0.56]
Ribeiro et al., 2019	0.89 [0.81, 0.93]	0.64 [0.53, 0.74]
Ribeiro et al., 2019	0.79 [0.70, 0.85]	0.84 [0.74, 0.90]
Ribeiro et al., 2019	0.92 [0.86, 0.96]	0.88 [0.78, 0.93]
Siripattanapipong et al., 2017	0.89 [0.70, 0.97]	0.95 [0.68, 1.00]
Siripattanapipong et al., 2017	0.41 [0.24, 0.61]	0.77 [0.48, 0.93]
Siripattanapipong et al., 2017	0.23 [0.11, 0.43]	0.95 [0.68, 1.00]
Siripattanapipong et al., 2017	0.20 [0.08, 0.40]	0.95 [0.68, 1.00]
Khan et al., 2012	0.98 [0.92, 1.00]	1.00 [0.95, 1.00]
Rezaei et al., 2021	0.77 [0.68, 0.84]	0.98 [0.95, 0.99]
Sundar et al., 2007	1.00 [0.98, 1.00]	0.97 [0.91, 0.99]
Sundar et al., 2007	1.00 [0.98, 1.00]	0.85 [0.75, 0.92]
Sundar et al., 2007	0.23 [0.18, 0.29]	1.00 [0.95, 1.00]
Sundar et al., 2007	0.23 [0.18, 0.29]	0.99 [0.94, 1.00]
Khan et al., 2014	0.98 [0.90, 0.99]	0.94 [0.86, 0.98]

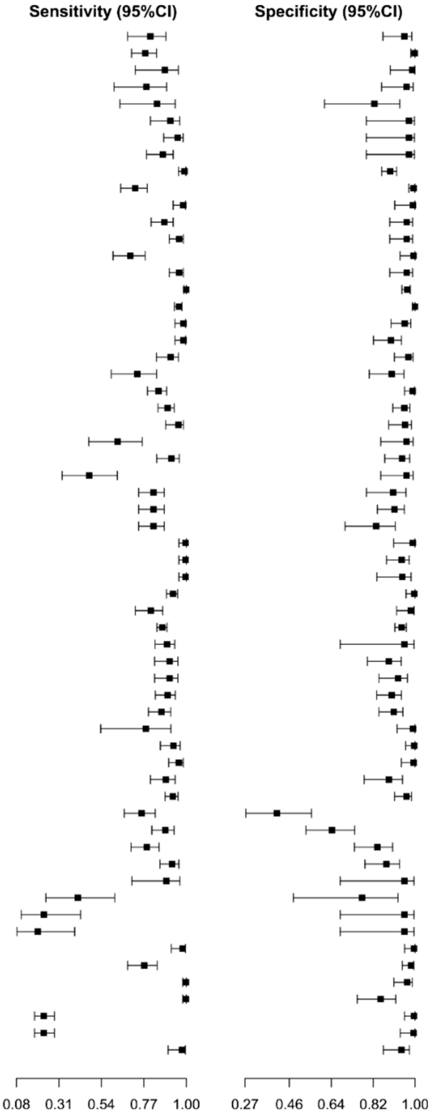

Figure S25. Study data and paired forest plots of the sensitivity and specificity of rapid diagnostic tests (RDT) in visceral leishmaniasis diagnosis. Data from each included study [98,102,116,117,122,125,127–130,132–135,137,140,141,146,158,180,184–189,191–197] are summarized. Sensitivity and specificity are reported with a mean (95% confidence limits). The forest plot depicts the estimated sensitivity and specificity (black squares) and their 95% confidence limits (horizontal black line).

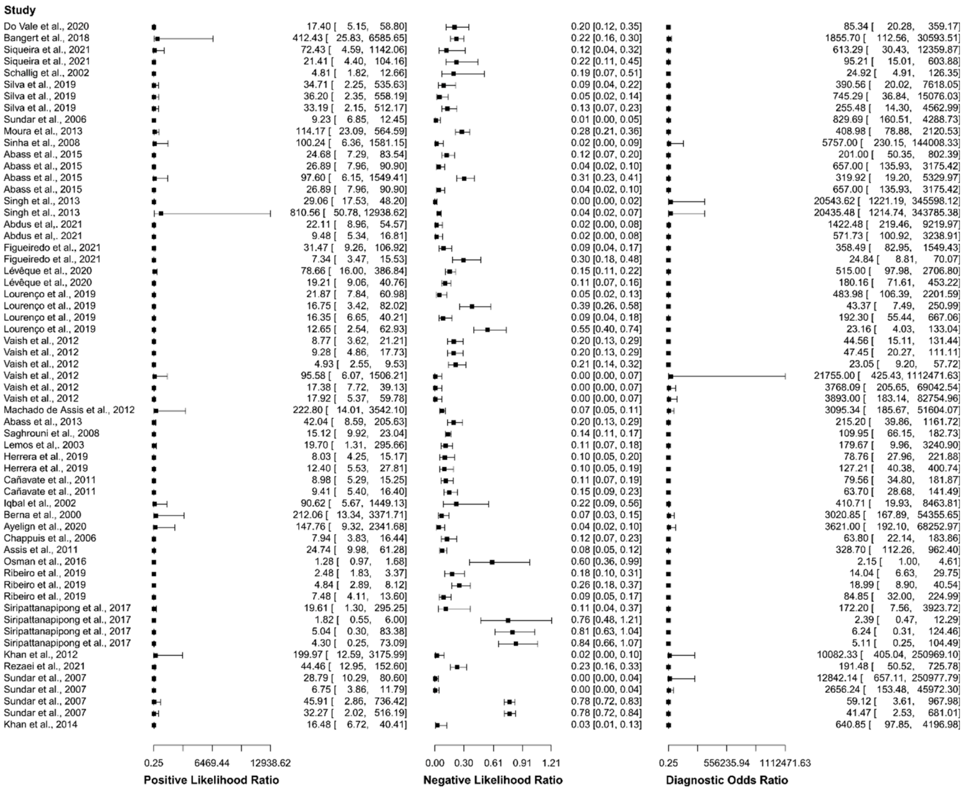

Figure S26. Study data and paired forest plots of the positive likelihood ratio, negative likelihood ratio, and diagnostic odds ratio of rapid diagnostic tests (RDT) in the diagnosis of visceral leishmaniasis. The positive likelihood ratio, negative likelihood ratio, and diagnostic odds ratio are reported with a mean (95% confidence limits) for the included studies [98,102,116,117,122,125,127–130,132–135,137,140,141,146,158,180,184–189,191–197].

Table S1. PRISMA 2020 Checklist

Section and Topic	Item #	Checklist item	Location where item is reported
**TITLE**			
Title	1	Identify the report as a systematic review.	Yes, Title
**ABSTRACT**			
Abstract	2	See the PRISMA 2020 for Abstracts checklist.	Yes
**INTRODUCTION**			
Rationale	3	Describe the rationale for the review in the context of existing knowledge.	Yes, Introduction Paragraph 1
Objectives	4	Provide an explicit statement of the objective(s) or question(s) the review addresses.	Yes, Introduction Paragraph 5
**METHODS**			
Eligibility criteria	5	Specify the inclusion and exclusion criteria for the review and how studies were grouped for the syntheses.	Yes, Selection criteria and data extraction
Information sources	6	Specify all databases, registers, websites, organizations, reference lists and other sources searched or consulted to identify studies. Specify the date when each source was last searched or consulted.	Yes, Search strategy
Search strategy	7	Present the full search strategies for all databases, registers and websites, including any filters and limits used.	Yes, Search strategy
Selection process	8	Specify the methods used to decide whether a study met the inclusion criteria of the review, including how many reviewers screened each record and each report retrieved, whether they worked independently, and if applicable, details of automation tools used in the process.	Yes, Selection criteria and data extraction
Data collection process	9	Specify the methods used to collect data from reports, including how many reviewers collected data from each report, whether they worked independently, any processes for obtaining or confirming data from study investigators, and if applicable, details of automation tools used in the process.	Yes, Selection criteria and data extraction
Data items	10a	List and define all outcomes for which data were sought. Specify whether all results that were compatible with each outcome domain in each study were sought (e.g. for all measures, time points, analyses), and if not, the methods used to decide which results to collect.	Yes, Selection criteria and data extraction
	10b	List and define all other variables for which data were sought (e.g. participant and intervention characteristics, funding sources). Describe any assumptions made about any missing or unclear information.	Yes, Selection criteria and data extraction
Study risk of bias assessment	11	Specify the methods used to assess risk of bias in the included studies, including details of the tool(s) used, how many reviewers assessed each study and whether they worked independently, and if applicable, details of automation tools used in the process.	Not applicable
Effect measures	12	Specify for each outcome the effect measure(s) (e.g. risk ratio, mean difference) used in the synthesis or presentation of results.	Not applicable
Synthesis methods	13a	Describe the processes used to decide which studies were eligible for each synthesis (e.g. tabulating the study intervention characteristics and comparing against the planned groups for each synthesis (item #5)).	Yes, Data collection and management
	13b	Describe any methods required to prepare the data for presentation or synthesis, such as handling of missing summary statistics, or data conversions.	Yes, Data collection and management
	13c	Describe any methods used to tabulate or visually display results of individual studies and syntheses.	Yes, Data collection and

Section and Topic	Item #	Checklist item	Location where item is reported
			management
	13d	Describe any methods used to synthesize results and provide a rationale for the choice(s). If meta-analysis was performed, describe the model(s), method(s) to identify the presence and extent of statistical heterogeneity, and software package(s) used.	Yes, Statistical analysis
	13e	Describe any methods used to explore possible causes of heterogeneity among study results (e.g. subgroup analysis, meta-regression).	Yes, Statistical analysis
	13f	Describe any sensitivity analyses conducted to assess robustness of the synthesized results.	Yes, Statistical analysis
Reporting bias assessment	14	Describe any methods used to assess risk of bias due to missing results in a synthesis (arising from reporting biases).	Not applicable
Certainty assessment	15	Describe any methods used to assess certainty (or confidence) in the body of evidence for an outcome.	Not applicable
**RESULTS**			
Study selection	16a	Describe the results of the search and selection process, from the number of records identified in the search to the number of studies included in the review, ideally using a flow diagram.	Yes, Results
	16b	Cite studies that might appear to meet the inclusion criteria, but which were excluded, and explain why they were excluded.	Yes, Results
Study characteristics	17	Cite each included study and present its characteristics.	Yes, Results
Risk of bias in studies	18	Present assessments of risk of bias for each included study.	Not applicable
Results of individual studies	19	For all outcomes, present, for each study: (a) summary statistics for each group (where appropriate) and (b) an effect estimates and its precision (e.g. confidence/credible interval), ideally using structured tables or plots.	Yes, Results
Results of syntheses	20a	For each synthesis, briefly summarize the characteristics and risk of bias among contributing studies.	Yes, Results
	20b	Present results of all statistical syntheses conducted. If meta-analysis was done, present for each the summary estimate and its precision (e.g. confidence/credible interval) and measures of statistical heterogeneity. If comparing groups, describe the direction of the effect.	Yes, Results
	20c	Present results of all investigations of possible causes of heterogeneity among study results.	Yes, Results
	20d	Present results of all sensitivity analyses conducted to assess the robustness of the synthesized results.	Yes, Results
Reporting biases	21	Present assessments of risk of bias due to missing results (arising from reporting biases) for each synthesis assessed.	Not applicable
Certainty of evidence	22	Present assessments of certainty (or confidence) in the body of evidence for each outcome assessed.	Not applicable
**DISCUSSION**			
Discussion	23a	Provide a general interpretation of the results in the context of other evidence.	Yes, Discussion
	23b	Discuss any limitations of the evidence included in the review.	Yes, Discussion
	23c	Discuss any limitations of the review processes used.	Yes, Discussion
	23d	Discuss implications of the results for practice, policy, and future research.	Yes, Discussion
**OTHER INFORMATION**			
Registration and protocol	24a	Provide registration information for the review, including register name and registration number, or state that the review was not registered.	Yes, Study protocol
	24b	Indicate where the review protocol can be accessed, or state that a protocol was not prepared.	Yes, Study protocol
	24c	Describe and explain any amendments to information provided at registration or in the protocol.	Yes, Study protocol
Support	25	Describe sources of financial or non-financial support for the review, and the role of the funders or sponsors in the review.	Yes, Grant information
Competing interests	26	Declare any competing interests of review authors.	Yes, Competing

Section and Topic	Item #	Checklist item	Location where item is reported
			interests
Availability of data, code and other materials	27	Report which of the following are publicly available and where they can be found: template data collection forms; data extracted from included studies; data used for all analyses; analytic code; any other materials used in the review.	Yes, Dataset availability statement

From: Page MJ, McKenzie JE, Bossuyt PM, Boutron I, Hoffmann TC, Mulrow CD, et al. The PRISMA 2020 statement: an updated guideline for reporting systematic reviews. BMJ 2021;372:n71. doi: 10.1136/bmj.n71. For more information, visit: **http://www.prisma-statement.org/**

Table S2. Main methodological aspects of studies on tegumentary leishmaniasis

Reference	Country	Diagnostic Test	Leishmania species	Sample size	Type of sample	Study design	Reference test
Gomes et al., 2014	Brazil	LST, ELISA, IFAT, PCR	*Leishmania (Viannia) braziliensis*	Cases: 17 patients with mucocutaneous leishmaniasis (ML), 19 patients with cutaneous leishmaniasis (CL). Controls: 33 patients with non-leishmaniasis disease.	Saliva, nasal swabs, and oral filter paper imprints	Case-control study	PCR on lesion biopsy imprints, smears, in vitro cultures, the Montenegro skin test, and indirect immunofluorescence
Soares et al., 2015	Brazil	LST, PCR	*Leishmania (Leishmania) mexicana*	Cases: 98 patients with American Tegumentary Leishmaniasis. Controls: 80 healthy individuals, 24 with Chagas disease, 13 with pemphigus foliaceus, 8 with leprosy, 9 with deep mycosis, 16 VDRL-positive patients, and 33 with rheumatic diseases .	Serum	Cross-sectional study	Direct examination, culture, histopathological examination, PCR from lesion fragments, MST (Montenegro skin test), and IFAT (immunofluorescence antibody test)
Garcia et al., 2007	Bolivia	LST, PCR	*Leishmania (Viannia) braziliensis Leishmania (Viannia) lainsoni*	Cases: 44 patients with confirmed American tegumentary leishmaniasis. Controls: 9 patients with non-leishmaniasis disease.	Skin scrapings, syringe aspirates, biopsies	Cross-sectional study	PCR-based method targeting the hsp70 gene with RFLP analysis
Neitzke-Abreu et al., 2013	Brazil	LST, IFAT, PCR	*Leishmania (Viannia) braziliensis*	Cases: 106 patients diagnosed with cutaneous leishmaniasis. Controls: 223 patients were included initially for suspicion of cutaneous leishmaniasis.	Lesion scarification and peripheral blood enriched with leukocytes	Cross-sectional study	Microscopy
Gomes et al., 2008	Brazil	LST	*Leishmania (Viannia) braziliensis complex*	Cases: 52 patients with cutaneous leishmaniasis. Controls: 57 patients with other etiologies (non-leishmaniasis disease).	Biopsies from cutaneous lesions	Observational study	Clinical features and at least one positive result from parasitological methods, PCR, or histopathological examination
Carvalho et al., 2017	Brazil	ELISA	*Leishmania braziliensis, Leishmania infantum*	Cases: 57 patients (30 with mucosal leishmaniasis (ML), 27 with cutaneous leishmaniasis (CL)). Controls: 40 healthy	Serum	Case-control study	Clinical evaluation, PCR for Leishmania braziliensis kDNA

				individuals and 15 patients with Chagas disease.			
Pedras et al., 2008	Brazil	ELISA	*Leishmania chagasi*	Cases: 88 patients with tegumentary leishmaniasis.<br>Controls: 20 non-infected individuals and 85 patients with other infectious diseases (30 with Chagas disease, 20 with malaria, 20 with syphilis, and 15 with schistosomiasis).	Serum	Case-control study	Indirect fluorescent antibody test
Szargiki et al., 2009	Brazil	ELISA, IFAT, WB	*Leishmania (Viannia) braziliensis, Leishmania (Leishmania) amazonensis*	Cases: 87 patients (69 with confirmed parasitological diagnosis).<br>Controls: 13 individuals from non-endemic areas without clinical signs and 51 individuals with other diseases (30 with paracoccidioidomycosis, 10 with Chagas' disease, and 8 with toxoplasmosis).	Serum	Observational study	Parasitological diagnosis (smear and/or culture)
Salotra et al., 2003	India	ELISA	*Leishmania donovani*	Cases: 25 patients with confirmed post-kala-azar dermal leishmaniasis (PKDL).<br>Controls: 25 controls, including 10 patients with lepromatous leprosy and 15 healthy individuals.	Skin biopsies, serum samples	Observational study	PCR
Duarte et al., 2015	Brazil	ELISA	*Leishmania (Viannia) braziliensis*	Cases: 43 patients with confirmed tegumentary leishmaniasis, including cutaneous and mucosal forms.<br>Controls: 30 non-infected individuals	Serum	Case-control study	Microscopic examination, the Montenegro skin test, and PCR
Cataldo et al., 2010	Brazil	ELISA	*Leishmania (Viannia) braziliensis*	Cases: 76 patients with confirmed tegumentary leishmaniasis.<br>Controls: 76 non-infected individuals.	Serum	Case-control study	Imprint, culture, or histopathology methods
Souza et al., 2013	Brazil	ELISA	*Leishmania infantum-chagasi*	Cases: 102 (53 with mucosal leishmaniasis and 49 with cutaneous leishmaniasis).<br>Controls: 88 (39 from	Serum	Case-control study	ELISA with soluble leishmania antigen

				endemic areas and 49 from non-endemic areas).			
Montoya et al., 1997	Peru and Colombia	ELISA	*Leishmania (Viannia) peruviana*	Cases: 78 human sera from patients diagnosed with Latin American tegumentary leishmaniasis.<br>Controls: 39 sera from individuals with other diseases and 10 negative controls from healthy individuals.	Serum	Case-control study	ELISA and Western blotting using whole Leishmania parasite extracts and recombinant antigens
Redhu et al., 2006	India	ELISA	*Leishmania donovani*	Cases: 50 parasitologically confirmed patients with leishmaniasis.<br>Controls: 50 healthy controls and 150 patients with other diseases.	Serum	Case-control study	rKE-16 ELISA and Rapid Immunodot test (Signal KA), using the Ld-rKE-16 recombinant antigen
Mosleh et al., 1995	Jordan	ELISA, IFAT	*Leishmania major*	Cases: 100 (37 parasitologically-proven, 42 with clinically-typical lesions but negative parasitological tests, 21 clinically-suspected cases).<br>Controls: 132 healthy blood donors, 10 patients with pulmonary tuberculosis, and 16 patients with typhoid fever.	Serum	Case-control study	IFAT and ELISA
Zeyrek et al., 2007	Turkey	ELISA, WB	*Leishmania tropica*	Cases: 51 untreated Anthroponotic Cutaneous Leishmaniasis patients + 62 treated Anthroponotic Cutaneous Leishmaniasis patients (total 113 ACL patients).<br>Controls: 29 visceral leishmaniasis patients (positive controls) + 43 blood donors (negative controls).	Serum	Comparative and observational study	WB
Rodrigues et al., 2019	Brazil	ELISA	*Leishmania braziliensis*	Cases: 59 cases (20 cutaneous leishmaniasis, 39 mucosal leishmaniasis.	Serum	Cross-sectional study	ELISA for evaluating antibody detection against recombinant Prohibitin and

				Controls: 45 non-infected.			synthetic peptide antigens
Deepachandi et al., 2020	Sri Lanka	ELISA	*Leishmania donovani*	Cases: 200 patients confirmed for cutaneous leishmaniasis. Controls: 200 individuals divided into groups: 50 endemic healthy controls, 50 non-endemic healthy controls, 50 patients with other skin diseases, and 50 patients with systemic diseases.	Serum	Case-control study	Light microscopy and in vitro culturing
Ribeiro et al., 2018	Brazil	ELISA	*Leishmania infantum, Leishmania braziliensis*	Cases: 45 (15 visceral leishmaniasis, 15 cutaneous leishmaniasis, 15 mucosal leishmaniasis). Controls: 90 (20 from endemic areas, 20 from non-endemic areas, and 50 with other diseases).	Serum	Case-control study	Parasitological diagnosis confirmed by PCR and additional serological tests
Pedrosa et al., 2017	Brazil	ELISA	*Leishmania braziliensis*	Cases: 45 patients (20 with cutaneous leishmaniasis and 25 with mucosal leishmaniasis). Controls: 50 healthy individuals (25 from endemic and 25 from non-endemic areas).	Serum	Case-control study	ELISA using soluble Leishmania braziliensis antigenic preparation
Massae et al., 2017	Brazil	ELISA	*Leishmania (Viannia) braziliensis, Leishmania (Leishmania) amazonensis, Leishmania (Viannia) guyanensis, Leishmania (Viannia) shawi*	Cases: 219 ATL patients. Controls: 68 healthy individuals and 213 other diseases (non-*Leishmania* conditions).	Serum	Observational, with both cross-sectional and comparative study	Recombinant antigens in ELISA tests, validated with direct parasitological exams, histopathology, and PCR
Bracamonte et al., 2020	Argentina	ELISA	*Leishmania (Viannia) braziliensis, Leishmania (Viannia) guyanensis*	Cases: 99 ATL diagnosed patients. Controls: 27 non-ATL patients and 84 donors from non-endemic areas.	Serum	Prospective study	Dermal smear microscopic examination, PCR, Leishmanin skin test, and clinical assessment
Garcia-Miss et al., 1990	Mexico	ELISA	*Leishmania mexicana mexicana*	Cases: 74 sera from patients with chiclero's ulcer.	Serum	Case-control study	ELISA for immunoglobulin G (IgG) antibodies

				Controls: 75 sera from healthy individuals with negative Montenegro tests. 56 sera from healthy individuals with positive Montenegro tests. 18 sera from patients with other diseases (e.g., Chagas disease, toxoplasmosis, malaria, mycosis, carcinoma).			
Menezes-Souza et al., 2014	Brazil	ELISA	*Leishmania (Viannia) braziliensis*	Cases: 65 samples (45 cutaneous leishmaniasis and 20 mucosal leishmaniasis). Controls: 50 samples from healthy individuals, 20 from Chagas disease patients.	Serum	Case-control study	Microscopy and PCR
Pedras et al., 2003	Brazil	IFAT	*Leishmania braziliensis, Leishmania amazonensis*	Cases: 36 patients (17 with mucosal leishmaniasis and 19 with muco-cutaneous leishmaniasis). Controls: 20 individuals for each of the following groups: Chagas disease, malaria, syphilis, and non-infected individuals.	Serum	Case-control study	ELISA and IFAT
Rocha et al., 2006	Brazil	IFAT	*Leishmania (Viannia) braziliensis*	Cases: 78 individuals with localized cutaneous leishmaniasis. Controls: 170 healthy individuals and individuals with other diseases like visceral leishmaniasis and Chagas disease.	Serum	Case-control study	IFAT
Gonçalves et al., 2002	Brazil	WB	*Leishmania (Viannia) braziliensis, Leishmania (Leishmania) amazonensis, Leishmania (Leishmania) tropica*	Cases: 108 patients with ATL. Controls: 23 chagasic patients, 32 patients with other diseases, 78 healthy individuals.	Serum	Case-control study	WB using antigens from promastigote forms of Leishmania and a trypanosomatid strain (268T)
Azmi et al., 2011	Palestinian Territories	PCR	*Leishmania tropica and*	Cases: 170 true positives.	Tissue aspirates	Retrospective analysis	At least two PCR assays or culture positivity

			Leishmania major	Controls: 42 true negatives.	and scrapings		
Fagundes et al., 2010	Brazil	PCR	*Leishmania (Viannia) braziliensis*	Cases: 130 patients with confirmed ATL. Controls: 15 patients with other diagnoses and 23 patients with lesions suggestive of ATL but no parasitological confirmation.	Biopsy fragments	Prospective analysis	Combination of imprint, histopathology, and culture
Cruz et al., 2002	Spain	PCR	*Leishmania infantum*	Cases: 38 patients confirmed as having leishmaniasis. Controls: 40 samples (20 from healthy volunteers and 20 from individuals with protozoal infections other than leishmaniasis).	Blood and bone marrow	Prospective study	Microscopy and culture
Lemrani et al., 2009	Morocco	PCR	*Leishmania infantum, Leishmania tropica Leishmania major*	Cases: 26 patients clinically suspected of cutaneous leishmaniasis. Controls: 5 patients with similar lesions but other conditions such as leprosy, psoriasis, etc.	Skin biopsies	Prospective study	Smear microscopy and in vitro culture
Srivastava et al., 2011	India	PCR	*Leishmania donovani*	Cases: 25 Post Kala-azar Dermal Leishmaniasis patients. Controls: 750 individuals (250 healthy controls from endemic regions, 250 healthy controls from non-endemic regions, and 250 individuals with other (non-leishmaniasis) diseases like malaria and tuberculosis).	Peripheral blood	Case-control study	Parasitological confirmation via demonstration of parasites in giemsa-stained smears
Isaza et al., 2002	Colombia	PCR	*Leishmania Viannia (specific species not detailed)*	Cases: 67 patients with confirmed cutaneous leishmaniasis. Controls: 21 patients with lesions of other etiologies.	Skin scrapings and biopsies	Case-control study	Conventional parasitological methods (scraping, culture, biopsy) compared to PCR using Bl/B2 primers
Mouttaki et al., 2014	Morocco	PCR	*Leishmania major, Leishmania tropica*	Cases: 44 confirmed positives. Controls: 14 true negatives.	Dermal syringe-sucked fluid	Prospective study	PCR-based assays (ITS1 PCR-RFLP identified species for all true positives, considered the most

			Leishmania infantum				accurate method in the study).
Muñoz et al., 2016	Ecuador	PCR	*Leishmania guyanensis, Leishmania shawi, Leishmania naiffi*	Cases: 20 patients were confirmed positive for leishmaniasis.<br>Controls: Data is not provided explicitly.	Whole blood and skin samples	Case-control study	Microscopic examination
Disch et al., 2005	Brazil	PCR	*Leishmania (Viannia) braziliensis, Leishmania (Viannia) colombiensis, Leishmania (Viannia) guyanensis*	Cases: 13 patients with mucosal leishmaniasis.<br>Controls: 10 patients with other chronic inflammatory mucosal diseases.	Mucosal tissue fragments	Cross-sectional study	PCR amplification of Leishmania genus and Viannia subgenus kDNA, alongside parasitological and clinical evaluations
Gomes Rodrigues et al., 2002	Brazil	PCR	*Leishmania (Viannia) braziliensis*	Cases: 88 patients with confirmed ACL.<br>Controls: 31 patients with non-leishmaniasis cutaneous lesions.	Skin biopsy specimens	Case-control study	Microscopic smear examination, Histopathological examination, Isolation by culture, and Detection of circulating antibodies using indirect immunofluorescence
Mathis et al., 1995	Switzerland	PCR	*Leishmania braziliensis, Leishmania infantum, Leishmania donovani, Leishmania tropica, Leishmania aethiopica, Leishmania major*	Cases: 96 confirmed positives.<br>Controls: 70 true negatives.	Skin biopsies, bone marrow biopsies, whole blood (leukocyte fraction)	Cross-sectional study	In vitro cultivation
Gangneux et al., 2003	France	PCR	*Leishmania major, Leishmania tropica, Leishmania infantum, Leishmania donovani, Leishmania archibaldi*	Cases: 29 positive samples.<br>Controls: 139 negative samples.	Bone marrow aspirates, blood samples, dermal scrapings, and biopsy specimens	Bone marrow aspirates, blood, dermal scrapings, and biopsy specimens	Combination of direct microscopic examination, in vitro culture, and PCR amplification with subsequent DNA sequencing.
Salotra et al., 2001	India	PCR	*Leishmania donovani*	Cases: 51 patients with kala-azar and 48 patients with post-kala-azar dermal leishmaniasis.	Blood, bone marrow, and skin lesions	Retrospective analysis	Microscopy and culture

				Controls: 81 total (15 malaria, 15 tuberculosis, 32 leprosy patients, and 20 healthy volunteers from endemic areas).			
Boni et al., 2017	Brazil	PCR, qPCR	*Leishmania braziliensis*	Cases: 25 patients with confirmed ATL. Controls: 10 healthy volunteers.	Biopsy samples and mucous swab samples	Observational study	PCR-kDNA
Montalvo et al., 2017	Colombia and Cuba	PCR	*Leishmania braziliensis, Leishmania donovani*	Cases: 90 patients with cutaneous leishmaniasis. Controls: 37 individuals.	Lesion scrapings or biopsies	Case-control study	PCR-18S, PCR-hsp70-N, and parasitological tests
Deepachandi et al., 2019	Sri Lanka	PCR	*Leishmania donovani*	Cases: 30 patients with cutaneous leishmaniasis. Controls: 30 individuals (10 with non-leishmanial skin diseases, 10 with other systemic diseases, and 10 healthy individuals)	Lesion material, skin biopsies, bone marrow aspirates, peripheral blood, and skin materials	Cross-sectional study	Nested PCR, light microscopy, in-vitro culture (IVC), and conventional single-step PCR
Ovalle-Bracho et al., 2016	Colombia	PCR	*Leishmania panamensis, Leishmania mexicana, Leishmania amazonensis, Leishmania infantum-chagasi*	Cases: 30 patients. Controls: 30 individuals.	Mucosal tissue biopsies	Case-control study	Clinical, epidemiological, and laboratory criteria
Safaei et al., 2002	Iran	PCR	*Leishmania tropica*	Cases: 62 (33 proven cases of cutaneous leishmaniasis and 29 clinically suspected but microscopically negative cases). Controls: 20 patients with confirmed skin diseases other than cutaneous leishmaniasis.	Skin biopsies	Retrospective analysis	Histological examination using microscopy
Ovalle Bracho et al., 2007	Colombia	PCR	*Leishmania braziliensis braziliensis, Leishmania mexicana amazonensis*	Cases: 36 patients. Controls: 25 individuals.	Biopsy samples	Case-control study	Clinical, histopathological, and therapeutic criteria

Gomes et al., 2017	Brazil	qPCR	*Leishmania (Viannia) braziliensis*	Cases: 55 patients with American tegumentary leishmaniasis (18 with mucosal leishmaniasis and 37 with cutaneous leishmaniasis). Controls: 36 patients without active American tegumentary leishmaniasis.	Swab and biopsy samples	Prospecti ve study	Montenegro skin test, serology, histopathology, smears, culture, and conventional PCR
Verma et al., 2013	India	qPCR	*Leishmania donovani*	Cases: 50 patients diagnosed with PKDL. Controls: 24 individuals (including cases of leprosy, sporotrichosis, and pityriasis lichenoides chronica).	Slit aspirate and tissue biopsy	Cross-sectional study	K39 serological strip test and qPCR
Morais et al., 2020	Brazil	qPCR	*Leishmania (Viannia) braziliensis, Leishmania (Viannia) guyanensis, Leishmania (Viannia) naiffi, Leishmania (Leishmania) amazonensis*	Cases: 213 patients. Controls: 23 individuals.	Blood samples, lesion biopsies, and lesion imprint on filter paper	Case-control study	Multilocus Enzyme Electrophoresis

Table S3. Main methodological aspects of studies on visceral leishmaniasis

Reference	Country	Diagnostic Test	Leishmania species	Sample size	Type of sample	Study design	Reference test
Bangert et al., 2018	Spain	DAT, IFAT, RDT	*Leishmania infantum*	Cases: 141 confirmed VL cases. Controls: 338 non-diseased individuals (including individuals with Chagas disease, malaria, other parasitic infections, and healthy blood donors).	Serum	Retrospective study	Nested PCR, Giemsa microscopy, and/or NNN culture from bone marrow or blood samples
Akhoundi et al., 2010	Iran	DAT	*Leishmania infantum*	Cases: 110 (7 confirmed cases and 103 clinically suspected cases) Controls: 218 (177 healthy individuals and 41 with other infectious diseases)	Serum	Comparative study	DAT
Oliveira et al., 2013	Brazil	DAT	*Leishmania infantum*	Cases: 103 serum samples from Brazilian patients with parasitologically confirmed visceral leishmaniasis. Controls: 110 samples, including individuals with other parasitic diseases (schistosomiasis, Chagas disease, malaria, tegumentary leishmaniasis) and healthy individuals.	Serum	Comparative study	DAT
Lita et al., 2002	Albania	DAT	*Leishmania infantum*	Cases: 50 children with confirmed visceral leishmaniasis Controls: 70 (40 healthy household contacts and 30 pediatric patients with other infections)	Sera and bone marrow aspirate	Prospective study	DAT

Osman et al., 2016	Sudan	DAT, RDT	*Leishmania donovani*	Cases: 96 visceral leishmaniasis patients<br>Controls: 42 (including malaria, tuberculosis, leukemia patients, and healthy blood donors).	Serum	Case-control study	Microscopy, DAT, and Rapid rK39 strip test
Oliveira et al., 2011	Brazil	DAT	*Leishmania chagasi*	Cases: 89 individuals with visceral leishmaniasis<br>Controls: 130 individuals (comprising patients with other diseases and healthy individuals)	Serum	Case-control study	DAT
Hasnain et al., 2014	Bangladesh	DAT	*Leishmania donovani*	Cases: 50 patients.<br>Controls: 50 individuals.	Venous blood	Case-control study	rK-39 strip test with response to anti-leishmanial treatment
Bimal et al., 2005	India	DAT	*Leishmania donovani*	Cases: 108 parasitologically confirmed cases of visceral leishmaniasis.<br>Controls: 641 (including 452 healthy individuals from non-endemic areas and 189 from areas adjacent to endemic regions).	Blood	Longitudinal study	DAT
el Harith et al., 1995	Bangladesh, Algeria, and Sudan	DAT	*Leishmania donovani, Leishmania infantum*	Cases: 86 patients with visceral leishmaniasis from Bangladesh (35), Algeria (24), and Sudan (20).<br>Controls: Healthy individuals (26), including Bangladesh (10), Sudan (8), Algeria (8), and additional samples from tuberculosis and malaria patients.	Serum	Case-control study	DAT

Oliveira et al., 2009	Brazil	DAT	*Leishmania (Leishmania) chagasi*	Cases: 61 patients with visceral leishmaniasis Controls: 96 individuals, including those with other diseases such as schistosomiasis, Chagas disease, and malaria.	Serum	Case-control study	DAT
Rijal et al., 2004	Nepal	DAT	*Leishmania donovani*	Cases: 155 parasitologically confirmed VL cases Controls: 77 non-VL cases	Urine	Prospective case-control study	Microscopic examination
Vogt et al., 2018	Ethiopia	DAT	*Leishmania donovani*	Cases: 87 patients with parasitologically confirmed VL Controls: not mentioned	Urine	Prospective study	Microscopic examination
Abdallah et al., 2004	Sudan	DAT	*Leishmania donovani*	Cases: 61 patients with microscopically confirmed VL Controls: 102 apparently healthy endemic controls, 8 patients with tuberculosis, 8 patients with malaria, and 8 patients with schistosomiasis	Serum and blood samples	Case-control study	Microscopy and PCR
Singla et al., 2003	India	DAT	*Leishmania donovani*	Cases: 58 confirmed VL Controls: 8 clinically suspected parasite-negative cases, 20 pulmonary tuberculosis cases, 14 malaria cases, 13 uninfected controls from endemic áreas, and 40 uninfected controls from non-endemic áreas.	Serum	Case-control study	DAT
Oskam et al., 1999	Ethiopia	DAT	*Leishmania donovani*	Cases: 203 active VL Controls: 2 relapse VL patients, 33	Serum	Comparative study	DAT

				treated VL patients, 9 HIV-VL co-infection, 36 localized cutaneous leishmaniasis, 7 diffuse cutaneous leishmaniasis, 1 HIV-CL co-infection, 100 patients with other diseases, 30 healthy controls.			
Meredith et al., 1995	Sudan	DAT	*Leishmania donovani*	Cases: 67 patients with parasitologically confirmed VL Controls: 52 healthy individuals from West Africa, 29 healthy individuals from areas in Sudan where VL is endemic, 354 individuals with diseases other than VL (including toxoplasmosis, malaria, and others).	Serum	Case-control study	DAT
Moody et al., 1996	Sudan	DAT	*Leishmania donovani*	Cases: 60 parasitologically proven cases Controls: 75 healthy controls	Serum	Case-control study	DAT
Schoone et al., 2001	Kenya, Sudan, and Ethiopia	DAT	*Leishmania donovani*	Cases (active visceral leishmaniasis): Group 1: 34 (Kenya, Sudan); Group 2: 205 (Ethiopia). Controls (healthy individuals): Group 1: 12 (Kenya, Sudan); Group 3: 32 (Côte d'Ivoire).	Serum and blood	Comparative study	DAT
Lourenço et al., 2019	Brazil	DAT, ELISA, IFAT, RDT	*Leishmania infantum*	Cases: 118 visceral leishmaniasis patients Controls: 118 non-diseased individuals	Serum	Comparative study	Microscopy and Culture
Machado de Assis et al., 2012	Brazil	DAT, ELISA, IFAT, RDT	*Leishmania (Leishmania) chagasi*	Cases: 285 visceral leishmaniasis patients	Bone marrow	Prospective study	Microscopy

				Controls: 119 non-diseased individuals	aspirates		
Abass et al., 2006	Sudan	DAT, ELISA	*Leishmania donovani*	Cases: 40 confirmed VL<br>Controls: 184 (including subgroups like malaria, typhoid, tuberculosis, and healthy individuals)	Serum, plasma, and whole-blood spotted	Case-control study	DAT
Bagchi et al., 1998	India	DAT, ELISA	*Leishmania donovani*	Cases: 51 parasitologically confirmed cases of VL<br>Controls: 25 healthy controls from non-endemic áreas, 26 endemic normal controls, and 103 patients suffering from other diseases (e.g., malaria, tuberculosis, AIDS).	Serum	Comparative study	DAT
Oliveira et al., 2017	Brazil	DAT	*Leishmania infantum*	Cases: 207 serum samples from patients with confirmed VL.<br>Controls: 80 serum samples, including individuals with other parasitic infections (19 with Chagas disease, 18 with schistosomiasis, 15 with tegumentary leishmaniasis) and 28 healthy individuals.	Serum	Case-control study	Microscopy and Culture
Kumar et al., 2006	India	DAT, ELISA	*Leishmania donovani*	Cases: 67 parasitologically confirmed patients with VL.<br>Controls: 431 healthy individuals from the study village, 10 healthy controls from an endemic area, and 40 individuals with other diseases (10 each for malaria, tuberculosis,	Serum	Case-control study	Microscopy

				leprosy, and typhoid).			
Abass et al., 2013	Sudan	DAT, ELISA, RDT	*Leishmania donovani*	Cases: 106 confirmed VL patients<br>Controls: 77 (30 healthy individuals from an endemic area, 20 from a non-endemic area, and 27 diseased controls including cases of malaria, tuberculosis, and leukemia)	Serum	Case-control study	DAT
Okong'o-Odera et al., 1993	Kenya	DAT, ELISA	*Leishmania donovani*	Cases: 8 patients confirmed with visceral leishmaniasis<br>Controls: 34 individuals from an endemic area and 68 former patients post-treatment	Serum	Case-control study	DAT
Attar et al., 2001	Brazil, Yemen, Nepal, Sudan	DAT	*Leishmania donovani*	Cases: 25 (Brazil), 5 (Nepal), 29 (Yemen), 73 (Sudan)<br>Controls: 34 endemic controls (Brazil), 23 endemic controls (Yemen), 312 non-endemic controls (Liverpool).	Urine	Case-control study	Microscopy
Cañavate et al., 2011	Ethiopia	DAT, IFAT, PCR, RDT	*Leishmania donovani*	Cases: 179 (125 suspected VL + 54 treated patients)<br>Controls: 67 healthy controls	Peripheral blood, spleen aspirates, serum, and whole blood	Case-control study	PCR, rK39 rapid tests, DAT, and IFAT
Chappuis et al., 2003	Nepal	DAT	*Leishmania donovani*	Cases: 139 patients with confirmed VL<br>Controls: 45 patients without VL	Serum	Prospective study	Microscopy
Bern et al., 2000	Nepal	DAT, RDT	*Leishmania donovani*	Cases: 92 individuals diagnosed with VL.<br>Controls: 113 individuals with no	Blood	Case-control study	Microscopy

				personal or household history of VL.			
do Vale et al., 2020	Brazil	DAT, IFAT, RDT	*Leishmania (Leishmania) infantum*	Cases: 53 individuals diagnosed with VL. Controls: 53 individuals with suspected VL but not confirmed.	Serum	Case-control study	Microscopy
Ayelign et al., 2020	Ethiopia	DAT, RDT	*Leishmania donovani*	Cases: 110 VL patients Controls: 162 (76 healthy controls and 86 with other diseases)	Serum	Case-control study	Microscopy
Schallig et al., 2002	Brazil	DAT, RDT	*Leishmania chagasi*	Cases (confirmed VL patients): 21 serum samples, 15 blood samples Controls (healthy and with other diseases): 19 healthy controls and 42 with other diseases	Serum and blood	Case-control study	DAT
Ben-Abid et al., 2017	Tunisia	DAT	*Leishmania infantum*	Cases: 35 VL patients Controls: 62 (34 non-infectious disease controls, 28 infectious disease controls)	Urine and oral fluid	Case-control study	Microscopy
Silva et al., 2019	Brazil	DAT, ELISA, IFAT, RDT	*Leishmania infantum*	Cases: 51 patients (confirmed by parasite visualization in bone marrow aspirates) Controls: 33 individuals (15 with chronic Chagas' disease and 18 healthy individuals from non-endemic areas)	Serum	Case-control study	Microscopy
Sundar et al., 2006	India	DAT, RDT	*Leishmania donovani*	Cases: 150 parasitologically confirmed patients with VL. Controls: 358 (100 healthy individuals from non-endemic	Serum	Cross-sectional study	Microscopy

				regions, 153 healthy individuals from endemic regions, and 105 patients with other diseases)			
Sinha et al., 2008	India	DAT, RDT	*Leishmania donovani*	Cases: 91 individuals with confirmed VL. Controls: 50 healthy controls, and additional groups including 46 individuals with diseases other than VL.	Serum	Comparative study	Microscopy, DAT, and Rapid rK39 strip test
Abass et al., 2015	Sudan, India, and France	DAT, ELISA, RDT	*Leishmania donovani, Leishmania infantum*	Cases: 142 samples from confirmed VL cases, 11 samples from VL/HIV co-infection cases. Controls: 24 samples from symptomatic cases with unconfirmed VL diagnosis, 25 samples from asymptomatic individuals, 69 samples from healthy controls and individuals with other diseases (e.g., malaria, toxoplasmosis).	Serum	Comparative study	Microscopy and Culture
Mansour et al., 2007	Sudan	DAT, ELISA	*Leishmania donovani*	Cases: 322 patients suspected of VL. Controls: 56 healthy individuals from the endemic area and 38 healthy female medical students.	Serum	Case-control study	Microscopy and DAT
Chappuis et al., 2006	Nepal	DAT, RDT	*Leishmania donovani*	Cases: 85 parasitologically proven cases Controls: 57 healthy controls	Serum, urine, and bone marrow aspirates	Prospective study	Microscopy and DAT
Akhoundi et al., 2013	Iran	DAT	*Leishmania infantum*	Cases: 43 positive cases with VL Controls: 30 healthy patients, and 32	Serum	Case-control study	DAT

				patients with other infections.			
Salam et al., 2011	Bangladesh	DAT	*Leishmania donovani*	Cases: 36 parasitologically confirmed patients with VL.<br>Controls: 40 healthy individuals (20 from endemic zones and 20 from non-endemic zones).	Urine	Prospective study	Microscopy
de Assis et al., 2011	Brazil	DAT, RDT	*Leishmania (Leishmania) chagasi*	Cases: 213 parasitologically proven cases<br>Controls: 119 healthy controls	Bone marrow aspirates and peripheral blood	Prospective study	Microscopy
Sundar et al., 2007	India	DAT	*Leishmania donovani*	Cases: 282 (230 confirmed cases, 52 probable cases)<br>Controls: 170 (100 healthy endemic controls and 70 non-cases)	Serum, splenic smears, blood on filter paper, and urine	Cross-sectional study	Microscopy, clinical features and response to treatment for defining cases
Hossain et al., 2021	Bangladesh	ELISA, LAMP, qPCR	*Leishmania donovani*	Cases: 80 Visceral Leishmaniasis (VL) patients<br>Controls: 80 endemic healthy individuals	Whole blood, dried blood spots, and urine	Case-control study	Clinical symptoms and rK39 RDT
Salles et al., 2017	Brazil	ELISA	*Leishmania infantum*	Cases: 30 patients with symptomatic VL.<br>Controls: 27 healthy individuals from endemic areas and 30 from non-endemic areas.	Serum	Case-control study	PCR for Leishmania infantum kDNA
Machado et al., 2020	Brazil	ELISA	*Leishmania infantum*	Cases: 25 patients with VL<br>Controls: 25 healthy individuals from endemic areas	Serum	Case-control study	PCR for Leishmania infantum kDNA
Dias et al., 2018	Brazil	ELISA	*Leishmania infantum, Leishmania braziliensis*	Cases: 45 human cases of VL<br>Controls: 35 healthy individuals and 235 Chagas disease patients	Serum	Case-control study	PCR for Leishmania kDNA

Ribeiro Santos et al., 2019	Brazil	ELISA	*Leishmania infantum*	Cases: 70 L. infantum-infected symptomatic Controls: 20 with other diseases, 96 healthy individuals	Serum	Case-control study	Microscopy, culture, and PCR
Abdus Salam et al., 2021	Bangladesh	ELISA, LAMP, PCR, RDT	*Leishmania donovani*	Cases: 100 confirmed VL patients Controls: 100 individuals (30 healthy endemic, 30 healthy non-endemic, 40 disease controls)	Blood	Cross-sectional study	Microscopy
Saliba et al., 2019	Brazil	ELISA	*Leishmania infantum*	Cases: 124 serum samples with VL Controls: 185 serum samples of healthy individuals	Serum	Comparative study	Microscopy and clinical features
Costa et al., 2017	Brazil	ELISA	*Leishmania infantum*	Cases: 39 humans with VL Controls: 39 healthy humans from endemic areas, and 14 humans with Chagas disease.	Serum	Case-control study	PCR for Leishmania infantum kDNA
Oliveira-da-Silva et al., 2020	Brazil	ELISA	*Leishmania infantum*	Cases: 25 individuals diagnosed with VL. Controls: 25 healthy individuals from endemic regions	Serum	Case-control study	PCR for Leishmania infantum kDNA
Abeijon et al., 2020	Brazil, Kenya	ELISA	*Leishmania infantum, Leishmania donovani*	Cases: 24 urine samples from Brazil and 45 urine samples from Kenya. Controls: 35 healthy controls and additional controls from non-VL diseases (e.g., 6 with cutaneous leishmaniasis, 6 with Chagas disease, 6 with schistosomiasis, and 12 with tuberculosis).	Urine	Case-control study	rK39 RDT and DAT

Abeijon et al., 2019	Brazil, Kenya, India	ELISA	*Leishmania infantum, Leishmania donovani*	Cases: 24 samples from Brazil, 45 from Kenya, and 10 from India Controls: 40 samples from healthy individuals living in the same geographical areas as the cases.	Urine	Case-control study	rK39 RDT and microscopy
Braz et al., 2002	Brazil	ELISA	*Leishmania chagasi*	Cases: 120 patients confirmed VL Controls: 30 healthy individuals, 168 asymptomatic individuals (with positive delayed-type hypersensitivity test), and others as specified (e.g., 31 with tuberculosis, 13 with cutaneous leishmaniasis, and 14 with Chagas' disease)	Serum	Prospective study	rK39 RDT
Daprà et al., 2008	Italy	ELISA	*Leishmania infantum*	Cases: 327 parasitologically proven cases Controls: 1113 healthy controls	Serum	Comparative study	IFAT
Vallur et al., 2015	Sudan, Ethiopia, Bangladesh, and Brazil	ELISA	*Leishmania donovani, Leishmania infantum*	Cases: 43 samples from Brazil, 64 from Sudan, 46 from Ethiopia, and 13 from Bangladesh. Controls: 49 non-endemic controls, 10 endemic controls, and 30 individuals with other diseases (human African trypanosomiasis, tuberculosis, and malaria).	Urine	Comparative study	rK39 RDT and microscopy
Vaish et al., 2012	India	ELISA, RDT	*Leishmania donovani*	Cases: 252 parasitologically confirmed VL patients Controls: 103 endemic healthy controls, 95 non-endemic healthy controls, and 88	Serum	Prospective study	rK28 and rK39 ELISA and microscopy

				individuals with other infectious diseases			
Ghosh et al., 2016	Bangladesh	ELISA	*Leishmania donovani*	Cases: 87 patients with VL<br>Controls: 33 healthy endemic controls, 16 healthy non-endemic controls, 16 disease controls, and 16 tuberculosis patients.	Serum and urine	Case-control study	rK39 RDT and microscopy
Vaish et al., 2012b	India	ELISA	*Leishmania chagasi*	Cases: 114 parasitologically confirmed VL patients.<br>Controls: 47 healthy controls from non-endemic regions, 95 healthy controls from endemic regions, and 44 subjects with other diseases (e.g., tuberculosis, malaria, amebic liver abscess, typhoid, and dengue).	Saliva and serum	Prospective study	Microscopy
De Doncker et al., 2005	Nepal	ELISA, PCR	*Leishmania donovani*	Cases: 56 confirmed VL patients<br>Controls: 39 confirmed non-VL cases and 28 healthy human controls (from non-endemic areas)	Blood	Case-control study	Microscopy and PCR
Costa et al., 2012	Brazil	ELISA	*Leishmania infantum*	Cases: 28 individuals with active VL<br>Controls: 16 non-diseased individuals	Serum	Retrospective study	ELISA and IFAT
Maache et al., 2005	Spain (with additional samples from Peru, Chile, and France)	ELISA	*Leishmania donovani, Leishmania braziliensis*	Cases: 20 patients with visceral leishmaniasis (Spain), 4 patients with cutaneous leishmaniasis (*L. braziliensis*, Peru).<br>Controls: 10 healthy individuals (Spain), additional controls from patients with	Serum	Case-control study	Microscopy and PCR

				other diseases such as syphilis, tuberculosis, and toxoplasmosis.			
Abeijon et al., 2013	Brazil	ELISA	*Leishmania infantum*	Cases: 20 individuals with active VL Controls: 20 non-diseased individuals	Urine	Case-control study	Microscopy
Fonseca et al., 2014	Brazil	ELISA	*Leishmania infantum*	Cases: 80 individuals with active VL Controls: 10 non-diseased individuals	Serum	Case-control study	Microscopy and IFAT
Bandyopadhyay et al., 2004	India	ELISA	*Leishmania donovani*	Cases: 38 clinically confirmed active VL patients. Controls: 20 healthy individuals from endemic areas, 10 from non-endemic areas, and 8 individuals with cross-reactive diseases (4 with malaria and 4 with tuberculosis).	Serum	Case-control study	Microscopy and ELISA
Mohapatra et al., 2010	India	ELISA	*Leishmania chagasi, Leishmania donovani*	Cases: 55 confirmed VL patients Controls: 25 endemic controls	Serum	Case-control study	Microscopy
Galaï et al., 2011	Tunisia	ELISA, qPCR	*Leishmania infantum*	Cases: 37 patients with VL Controls: 40 healthy individuals	Oral fluid and blood	Case-control study	Microscopy
Barbosa-de-Deus et al., 2002	Brazil	ELISA	*Leishmania major*	Cases: 49 sera from patients with VL Controls: 169 sera from uninfected individuals	Serum	Cross-sectional study	IFAT
Islam et al., 2002	Bangladesh	ELISA	*Leishmania donovani*	Cases: 62 VL patients Controls: 214 non-VL individuals (including 59 healthy individuals from endemic areas, 53 healthy individuals from non-endemic areas,	Urine and serum	Comparative study	ELISA

				59 malaria patients, 13 tuberculosis patients, 23 cutaneous leishmaniasis patients, and 7 patients with other diseases)			
Kumar et al., 2011	India	ELISA	*Leishmania donovani*	Cases: 101 parasitologically confirmed VL patients. Controls: 93 nonendemic healthy control individuals, 110 endemic healthy control individuals, and 110 individuals with other diseases (e.g., amoebic liver abscess, tuberculosis, malaria).	Serum	Case-control study	rK39 RDT
Celeste et al., 2014	Brazil	ELISA	*Leishmania infantum*	Cases: 30 patients with VL. Controls: 30 healthy blood bank donors and 79 patients with other infectious diseases.	Serum	Case-control study	ELISA using Leishmania infantum rHsp83 antigen and total L. major-like promastigote antigen
Moreno et al., 2006	Brazil	ELISA, IFAT	*Leishmania (L.) chagasi*	Cases: 102 seropositive individuals (subset of initially 1,604 participants, with further investigation of 226 participants including 102 seropositive). Controls: 124 seronegative individuals (from the subset of 226 participants).	Blood	Cross-sectional study	DNA hybridization
Lakhal et al., 2012	Tunisia	ELISA	*Leishmania infantum*	Cases: 42 infantile VL patients. Controls: 70 matched control subjects (plus an additional group of 65 healthy women	Serum	Case-control study	Microscopy and qPCR

				for toxoplasmosis screening, used in secondary comparisons).			
Kumar et al., 2012	India	ELISA	*Leishmania donovani*	Cases: 108 parasitologically confirmed VL patients. Controls: 82 endemic healthy controls, 87 nonendemic healthy controls, and 77 individuals with different diseases (28 malaria, 25 tuberculosis, 15 viral fever, and 9 liver abscess).	Serum	Case-control study	WB and ELISA
Oliveira et al., 2011	Brazil	ELISA	*Leishmania infantum*	Cases: 39 patients with VL. Controls: 50 healthy patients and patients with other diseases (26 with cutaneous leishmaniasis, 40 with Chagas' disease)	Serum	Case-control study	ELISA using total Leishmania antigen and recombinant proteins
Kurkjian et al., 2005	Bangladesh	ELISA	*Leishmania donovani*	Cases: 33 patients with VL Controls: 4708 healthy individuals	Serum	Case-control study	rK39-based ELISA
Fargeas et al., 1996	Brazil, Sudan	ELISA	*Leishmania donovani*	Cases: 35 patients with VL. Controls: 17 healthy donors and 42 individuals with other infectious diseases (including 6 with cutaneous leishmaniasis, 14 with trypanosomiasis, 10 with strongyloidiasis, 10 with amoebiasis, and 8 with malaria).	Serum	Case-control study	DAT
Silva et al., 2011	Brazil	ELISA	*Leishmania chagasi*	Cases: 122 patients with VL Controls: 124 healthy individuals	Serum	Cross-sectional study	Montenegro skin test, ELISA, and IFAT

Kumar et al., 2011	India	ELISA	*Leishmania donovani*	Cases: 70 patients with parasitologically confirmed VL. Controls: 48 healthy individuals from endemic regions, 60 healthy individuals from non-endemic regions, and 42 individuals with other diseases (e.g., amoebic liver abscess, tuberculosis, malaria).	Serum	Case-control study	Microscopy
de Assis et al., 2009	Brazil	ELISA, PCR	*Leishmania chagasi*	Cases: 65 patients with VL Controls: 34 individuals (17 healthy and 17 with other febrile hepatosplenic diseases)	Serum	Case-control study	Microscopy and IFAT
Lemos et al., 2003	Brazil	ELISA, RDT	*Leishmania (Leishmania) chagasi*	Cases: 128 patients with confirmed parasitological diagnosis of VL Controls: 60 individuals (10 healthy and 50 with other infections: malaria, leprosy, Chagas disease, tuberculosis, or cutaneous leishmaniasis)	Serum	Case-control study	ELISA using soluble total antigen of Leishmania chagasi
Garcia et al., 2009	Brazil	ELISA	*Leishmania chagasi*	Cases: 35 patients with VL Controls: 20 cured VL patients and 60 control samples (10 healthy, and 10 each for Chagas disease, American Tegumentary Leishmaniasis, Tuberculosis, Malaria, and Hansen's disease)	Serum	Cross-sectional study	ELISA
Vallur et al., 2016	Bangladesh	ELISA	*Leishmania donovani*	Cases: 104 asymptomatic individuals patients with VL. Controls: 46 healthy	Serum	Cross-sectional study	DAT

				individuals (non-endemic controls) and 48 endemic healthy controls.			
Chauhan et al., 2016	India	ELISA	*Leishmania donovani*	Cases: 48 patients with VL<br>Controls: 20 healthy individuals	Serum and plasma	Case-control study	rK39-based ELISA
Siqueira et al., 2021	Brazil	ELISA, RDT	*Leishmania infantum*	Cases: 50 individuals with VL.<br>Controls: 22 healthy individuals and 54 patients chronically infected with *Trypanosoma cruzi* for cross-reactivity tests.	Serum	Comparative study	Microscopy and qPCR
Figueiredo et al., 2021	Brazil	ELISA, RDT	*Leishmania infantum*	Cases: 321 patients with VL<br>Controls: 409 healthy individuals	Serum	Prospective study	rK39-based ELISA, microscopy, and DAT
Lévêque et al., 2020	France, Tunisia, Morocco	ELISA, RDT	*Leishmania infantum*	Cases: 181 patients with VL<br>Controls: 138 healthy individuals	Serum	Retrospective study	WB
Iqbal et al., 2002	Kuwait	IFAT, RDT	*Leishmania donovani, Leishmania infantum*	Cases: 21 documented VL cases.<br>Controls: 75 healthy individuals and 155 patients with other parasitic infections.	Serum	Observational study	Microscopy
Saghrouni et al., 2009	Tunisia	IFAT, RDT	*Leishmania infantum, Leishmania major*	Cases: 574 Visceral Leishmaniasis patients<br>Controls: 355 (including 54 with cutaneous leishmaniasis, 42 with other protozoan infections, 152 with non-parasitic diseases, and 107 healthy individuals)	Serum	Retrospective study	IFAT, rK39-RDT
Rezaei et al., 2021	Iran	IFAT, qPCR, RDT	*Leishmania infantum*	Cases: 102 children diagnosed with VL.<br>Controls: 50 healthy children from endemic áreas, 46 healthy individuals	Blood	Retrospective study	qPCR, IFAT, and rK39-RDT

				from non-endemic áreas, and 47 individuals with non-VL diseases (e.g., malaria, cutaneous leishmaniasis).			
Gallardo et al., 1996	Spain	IFAT	*Leishmania infantum*	Cases: 14 patients with VL Controls: 106 healthy individuals	Serum and bone marrow aspirates	Case-control study	Microscopy
Abdus Salam et al., 2021	Bangladesh	ELISA, LAMP, PCR, RDT	*Leishmania donovani*	Cases: 100 confirmed VL patients Controls: 100 individuals (30 healthy endemic, 30 healthy non-endemic, 40 disease controls)	Blood	Cross-sectional study	Microscopy
Khan et al., 2012	Bangladesh	LAMP, PCR, RDT	*Leishmania donovani*	Cases: 75 parasitologically confirmed patients. Controls: 101 individuals (25 endemic healthy controls, 26 non-endemic healthy controls, 25 tuberculosis patients, and 25 with other febrile diseases).	Buffy coat from blood	Prospective study	Ln-PCR
Motazedian et al., 2008	Iran	PCR	*Leishmania infantum*	Cases: 30 confirmed patients with VL. Controls: 30 individuals (5 healthy, 15 with cutaneous leishmaniasis, 10 with malaria, brucellosis, or hydatid cyst).	Urine	Case-control study	Microscopy, IFAT, PCR
Khan et al., 2014	Bangladesh	PCR, RDT	*Leishmania donovani*	Cases: 61 confirmed VL patients Controls: 75 individuals (25 healthy individuals from endemic areas, 25 from non-endemic areas, and	Peripheral blood buffy coat	Observational case-control study	Microscopy

				25 with other diseases)			
Silva Pedrosa et al., 2013	Brazil	PCR	*Leishmania chagasi*	Cases: 47 patients with VL<br>Controls: 41 healthy individuals	Bone marrow aspirates and peripheral blood	Prospective study	Microscopy
Salam et al., 2010	Bangladesh	PCR	*Leishmania donovani*	Cases: 97 clinically suspected patients of VL.<br>Controls: 40 healthy individuals (20 from endemic and 20 from non-endemic areas).	Buffy coat from peripheral blood	Case-control study	Microscopy
Vaish et al., 2011	India	PCR	*Leishmania donovani*	Cases: 148 patients confirmed to have VL<br>Controls: 159 individuals (92 healthy subjects from endemic regions, 39 healthy subjects from non-endemic regions, and 28 individuals with other diseases)	Buccal swabs	Cross-sectional study	Microscopy
Molina et al., 2013	Spain	PCR	*Leishmania infantum*	Cases: 16 patients with VL<br>Controls: Not applicable	Peripheral blood and bone marrow	Prospective cohort study	Microscopy and Culture
Kaouech et al., 2008	Tunisia	PCR	*Leishmania infantum*	Cases: 53 pediatric patients with VL<br>Controls: 15 children with leukemia	Peripheral blood and bone marrow	Prospective study	Microscopy, culture, and PCR
Nuzum et al., 1995	India, Kenya, Brazil	PCR	*Leishmania donovani, Leishmania chagasi*	Cases: 63 patients before treatment (38 from India, 11 from Kenya, 10 from Brazil, and others unspecified).<br>Controls: 40 healthy individuals (27 Americans, 5 Indians, 7 Kenyans, 1 Brazilian).	Peripheral blood	Case-control study	Microscopy
Maurya et al., 2005	India	PCR	*Leishmania donovani*	Cases: 101 patients with parasitologically	Peripheral blood	Prospective study	Microscopy, culture, and

				proven VL. Controls: 150 individuals (50 healthy subjects from regions of endemicity, 50 from non-endemic regions, and 50 with other diseases).			PCR using Ld1 primers
Lachaud et al., 2000	France	PCR	*Leishmania infantum*	Cases: 36 patients diagnosed with Visceral Leishmaniasis MVL. Controls: 30 negative control patients without signs of disease.	Peripheral blood and bone marrow samples	Prospective study	Microscopy and Culture
Deborggraeve et al., 2008	Nepal	PCR	*Leishmania donovani, Leishmania infantum, Leishmania braziliensis, Leishmania peruviana, Leishmania guyanensis, Leishmania amazonensis, Leishmania panamensis, Leishmania lainsoni, Leishmania major, Leishmania aethiopica, Leishmania tropica*	Cases: 173 confirmed VL patients (140 blood and 170 bone marrow samples) Controls: 247 non-endemic control persons (19 healthy Belgian blood donors, 25 *T.b. gambiense* patients, and 230 *P. falciparum* patients from other regions)	Blood and bone marrow	Retrospective study	Microscopy
Disch et al., 2003	Brazil	PCR	*Leishmania (Leishmania) chagasi*	Cases: 53 patients with parasitologically-confirmed VL. Controls: 15 healthy, non-exposed volunteers.	Peripheral blood	Comparative study	Microscopy and Culture
Hossain et al., 2017	Bangladesh	PCR, qPCR	*Leishmania donovani*	Cases: 40 VL patients Controls: 80 endemic healthy controls, and 10 tuberculosis cases	Buffy coat from peripheral blood and skin biopsies	Cross-sectional study	rK39-RDT and Ln-PCR

Brustoloni et al., 2007	Brazil	PCR	*Not explicitly stated*	Cases: 91 patients with VL Controls: 79 individuals with other diseases or conditions	Bone marrow aspirate	Retrospective study	Microscopy, culture, and PCR
Basiye et al., 2010	Kenya	PCR	*Leishmania donovani*	Cases: 84 patients with confirmed VL Controls: 98 healthy endemic controls	Blood	Prospective study	Microscopy and DAT
Moura et al., 2013	Brazil	RDT	*Leishmania (Leishmania) infantum*	Cases: 145 patients with confirmed VL Controls: 236 healthy endemic controls	Serum	Observational study	IFAT
Singh et al., 2013	India	RDT	*Leishmania donovani*	Cases: 365 parasitologically confirmed VL patients Controls: 421 individuals (162 healthy persons from endemic areas, 154 healthy persons from non-endemic areas, and 105 patients with other infections)	Serum and urine	Case-control study	Microscopy
Herrera et al., 2019	Colombia	RDT	*Leishmania infantum, Leishmania amazonensis, Leishmania braziliensis*	Cases: 82 patients with confirmed VL Controls: 54 healthy endemic controls	Serum	Retrospective study	IFAT
Ribeiro Dos Santos et al., 2019	Brazil	RDT	*Leishmania infantum*	Cases: 70 patients with confirmed VL Controls: 20 other disease-infected, and 96 healthy individuals	Serum	Comparative study	Microscopy, culture, and PCR
Siripattanapipong et al., 2017	Thailand	RDT	*Leishmania martiniquensis*	Cases: 22 symptomatic VL patients Controls: 10 healthy individuals	Serum	Comparative study	rK39-RDT
Sundar et al., 2007	India	RDT	*Leishmania donovani*	Cases: 282 (230 confirmed cases + 52 probable cases) Controls: 170 (70 non-cases + 100	Serum, blood on filter paper, urine, and	Comparative study	DAT

		healthy endemic controls)	splenic smear	
